# Assessing the Potential of Untargeted SWATH Mass Spectrometry-Based Metabolomics to Differentiate Closely Related Exposures in Observational Studies

**DOI:** 10.3390/metabo12100942

**Published:** 2022-10-04

**Authors:** Frank Klont, Piotr Sosnowski, Daan Kremer, Tim J. Knobbe, Ron Bonner, Hans Blokzijl, Rinse K. Weersma, Stephan J. L. Bakker, TransplantLines Investigators, Eelko Hak, Daan J. Touw, Gérard Hopfgartner

**Affiliations:** 1 Life Sciences Mass Spectrometry, Department of Inorganic and Analytical Chemistry, University of Geneva, Quai Ernest Ansermet 24, 1211 Geneva, Switzerland; 2 Unit of PharmacoTherapy, -Epidemiology & -Economics, Groningen Research Institute of Pharmacy, University of Groningen, Antonius Deusinglaan 1, 9713 AV Groningen, The Netherlands; 3 Department of Clinical Pharmacy and Pharmacology, University Medical Center Groningen, University of Groningen, Hanzeplein 1, 9700 RB Groningen, The Netherlands; 4 Department of Internal Medicine, Division of Nephrology, University Medical Center Groningen, University of Groningen, Hanzeplein 1, 9700 RB Groningen, The Netherlands; 5 Ron Bonner Consulting, Newmarket, ON L3Y 3C7, Canada; 6 Department of Gastroenterology, University Medical Center Groningen, University of Groningen, Hanzeplein 1, 9700 RB Groningen, The Netherlands; 7 Group of Authors on Behalf of the Transplant Lines Biobank and Cohort Study, University Medical Center Groningen, University of Groningen, Hanzeplein 1, 9700 RB Groningen, The Netherlands; 8 Department of Pharmaceutical Analysis, Groningen Research Institute of Pharmacy, University of Groningen, Antonius Deusinglaan 1, 9713 AV Groningen, The Netherlands

**Keywords:** data-independent acquisition, exposomics, liquid chromatography, mass spectrometry, metabolomics, SWATH, transplantation

## Abstract

Mass spectrometry (MS) is increasingly used in clinical studies to obtain molecular evidence of chemical exposures, such as tobacco smoke, alcohol, and drugs. This evidence can help verify clinical data retrieved through anamnesis or questionnaires and may provide insights into unreported exposures, for example those classified as the same despite small but possibly relevant chemical differences or due to contaminants in reported exposure compounds. Here, we aimed to explore the potential of untargeted SWATH metabolomics to differentiate such closely related exposures. This data-independent acquisition MS-based profiling technique was applied to urine samples of 316 liver and 570 kidney transplant recipients from the TransplantLines Biobank and Cohort Study (NCT03272841), where we focused on the immunosuppressive drug mycophenolate, which is either supplied as a morpholino-ester prodrug or as an enteric-coated product, the illicit drug cocaine, which is usually supplied as an adulterated product, and the proton pump inhibitors omeprazole and esomeprazole. Based on these examples, we found that untargeted SWATH metabolomics has considerable potential to identify different (unreported) exposure or co-exposure metabolites and may determine variations in their abundances. We also found that these signals alone may sometimes be unable to distinguish closely related exposures, and enhancement of differentiation, for example by integration with pharmacogenomics data, is needed.

## 1. Introduction

Human health is influenced by multiple factors, including genetic predisposition, nutrition, lifestyle choices, environmental exposures, and medical care [1]. Due to the importance of these factors for our wellbeing, researchers worldwide are studying them intensively using a variety of research instruments. For example, genetic predisposition is frequently studied using sequencing technologies, lifestyle and nutrition are mostly queried through questionnaires, and assessment of environmental and medical care-related factors often relies on data mining, respectively utilizing environmental data registries and patient records [2,3,4,5,6].

For every health determinant, the availability and quality of research instruments is key to evaluating its importance. Taking genetic predisposition as an example, the understanding of causes and mechanisms of complex diseases remained limited prior to completion of the Human Genome Project (HGP) in 2003. Its completion, however, instigated the genome revolution which transformed biomedical sciences and put more emphasis on genetic factors as important health determinants [7]. The success story of genomics medicine furthermore stressed the need to keep advancing analytical technologies, since the HGP could not have achieved its goal in 2003 without the advent of capillary sequencing machines in the years before [8].

Despite the lessons learned from the HGP roughly twenty years ago, many health determinants are still studied using subjective instruments that rely on the principle of self-reporting, in particular in lifestyle, nutrition, and pharmacoepidemiologic research [9,10,11]. There is, however, a growing interest in bioanalytical approaches to yield more objective data in these different forms of exposure research [12,13,14]. A notable example in this regard is the use of mass spectrometry (MS)-based workflows to objectively determine exposures, such as habitual alcohol consumption through targeted quantification of the ethyl glucuronide metabolite in urine or illicit drug use through untargeted profiling of drugs of abuse in hair [15,16].

Untargeted profiling methods are particularly gaining momentum in clinical exposure research, as illustrated by several recent studies reporting discrepancies between self-reported and bioanalytical data on exposures [17,18,19,20]. The corresponding profiling methods have a very high identification capability for known compounds, and recent advances in data processing are facilitating the elucidation of increasing numbers of unknown chemicals [21]. Still, several challenges prevent untargeted profiling methods from reaching their full potential in clinical exposure research, including the ability to distinguish exposure to closely related compounds.

In this study, we explore the potential of the untargeted profiling technique ‘SWATH metabolomics’ to differentiate closely related exposures using almost 900 urine samples obtained from the TransplantLines Biobank and Cohort Study [22]. We selected three representative challenges for this purpose in order to emphasize both the compound identification and targeted signal extraction capabilities of this technique within the same analysis. Firstly, we studied usage of the immunosuppressive drug mycophenolate (MPA), which can be supplied either as a mofetil prodrug or as enteric-coated MPA. Secondly, we studied exposure to cocaine and possible cocaine adulterants. Thirdly, we studied the proton pump inhibitors omeprazole (i.e., equal mixture of R- and S-omeprazole) and esomeprazole (i.e., S-omeprazole). These examples represent analytically distinct and clinically relevant challenges, respectively, due to the varying degrees of closeness between the corresponding chemical exposures and the clinical importance of these chemicals in light of therapeutic efficacy and safety.

## 2. Materials and Methods

### 2.1. Clinical Samples

This study used 24-h urine samples from the TransplantLines Biobank and Cohort Study (NCT identifier NCT03272841), which was approved by the Institutional Review Board of the University Medical Center Groningen (UMCG; decision METc 2014/077) and adheres to the UMCG Biobank Regulation, the Declaration of Helsinki, and the Declaration of Istanbul [22]. The samples were collected (per strict protocol designed within the UMCG for generic biobanking purposes and lacking the addition of preservative agents commonly used in metabolomics research, such as protease inhibitors and boric acid) in BD Vacutainer 24-h urine collection containers (<48 h between sample collection and handing it in), and samples were stored at −20 °C for up to four days after manual aliquoting. Next, samples were stored at −80 °C and atmospheric pressure for up to five years until shipment (<72 h on dry ice in a security-sealed, insulated box compliant with IATA, ADR, and 49 CFR (DOT) transport regulations) and up to six months after shipment. For this study, we analyzed samples from 316 liver and 570 kidney transplant recipients who were ≥1 year post-transplantation and had already been transplanted prior to the start of the TransplantLines study.

### 2.2. Small-Molecule Profiling

After thawing (overnight at −25 °C, <4 h at 2–6 °C), vortex-mixing (30 s), and centrifugation (4 °C, 10 min, 14,000× *g*), 50 microliters of supernatant were transferred to glass inserts (BGB; Cat. No. 110501) placed in glass autosampler vials (BGB; Cat. No. SF2) and sealed with plastic caps (BGB; Cat. No. 070301). The urine was mixed with 10 microliters of a 5 pmol/µL internal standard solution in 10% methanol (see Table S1) by vortex-mixing (30 s). Next, 24 microliters of sample solution were analyzed by reversed-phase liquid chromatography coupled to high-resolution quadrupole-time-of-flight mass spectrometry operated in positive electrospray ionization and SWATH data-independent acquisition (DIA) modes. A detailed overview of LC and MS parameters is provided in Table S2.

### 2.3. Data Processing

Mycophenolate-positive samples were identified by spectral library matching [17] (SLM) using SCIEX PeakView software (version 2.2.0.11391; 71 Four Valley Drive, Concord, ON, Canada, L4K 4V8) and in-house generated reference spectra for glucuronidated mycophenolate (obtained with SCIEX TripleTOF instruments at a collision energy of 40 eV and a collision energy spread of 30 eV) followed by the feature-based evaluation of SLM results as presented in [19]. The same software and a commercial forensic MS/MS spectral library from SCIEX (version 1.1; 71 Four Valley Drive, Concord, ON, Canada, L4K 4V8; 1700 entries; obtained with SCIEX TripleTOF instruments at a collision energy of 35 eV and a collision energy spread of 15 eV) were used for identification of benzoylecgonine-positive samples, which was confirmed by sample reanalysis and by using a targeted assay [23], and for identifying possible adulterants in these samples. Omeprazole-positive samples were identified following SRM-like targeted signal extraction using SCIEX MultiQuant software (version 2.1) with a ± 2.5 mDa mass extraction window and a 2.0-point Gaussian smoothing width. Specifically, signals were extracted for five possible oxidation products of omeprazole, including its main metabolites, 5-hydroxyomeprazole and omeprazole sulfone (see Figures S1–S3). Here, a positive identification required signals above the detection limit, as was established according to the detection limit estimation approach presented in [19] for at least three metabolites. At last, feature-based analyses were performed using SCIEX MarkerView software (version 1.3.1; 71 Four Valley Drive, Concord, ON, Canada, L4K 4V8), and detailed overviews of data (pre)processing settings are provided in Tables S3 and S4.

## 3. Results and Discussion

### 3.1. Sample Analysis

**Figure 1:**
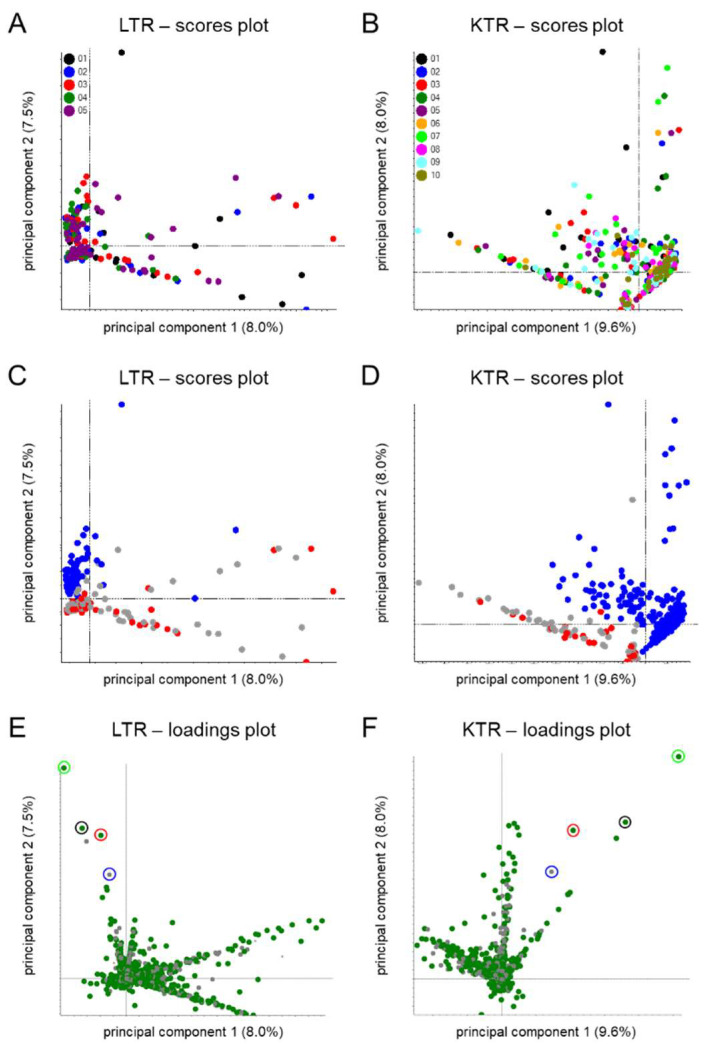
(**A**–**D**) Pareto-scaled scores and (**E**,**F**) loading plots for unsupervised principal component analysis of MS1-level feature data of stable (**A**,**C**,**E**) liver and (**B**,**D**,**F**) kidney transplant recipients. Different coloring was applied to the samples in the score plots based on (**A**,**B**) the analytical batches in which these were measured or (**C**,**D**) the use of azathioprine (in red) and mycophenolate (in blue). The latter was determined by spectral library matching using in-house generated reference spectra for 6-thiouric acid (Toronto Research Chemicals, Cat. No. T375500) and mycophenolate glucuronide (Toronto Research Chemicals, Cat. No. M831520), respectively. Coloring was also applied to the features in the loadings plots based on whether peaks were assigned as monoisotopic peaks (in green) or not (in gray). Furthermore, an unknown feature (*m*/*z* 610/8.4 min) which clusters around some mycophenolate-related features is indicated with a red circle in the loadings plots whereas its isotope peak is indicated with a blue circle. Features which correspond to the residual precursor and deglucuronidated version of mycophenolate glucuronide (as ammonium adduct) are indicated with green and black circles, respectively.

Urine samples were obtained from the TransplantLines Biobank and Cohort Study [22] for stable liver (LTR) and kidney transplant recipients (KTR). Included patients had a functional graft for at least 1 year post-transplantation, had already been transplanted before the biobank was started, and a sufficient amount of sample material had been biobanked (see Table S5). In total, 316 LTR samples were analyzed between November 18 and 22, 2021, and 570 KTR samples were analyzed between 24 November and 3 December 2021 in batches which were constructed following widely adopted recommendations [24]. Analytical performance was monitored using stable-isotope-labelled standards, as described previously [19], and a check for potential batch effects was performed using principal component analysis (PCA). The latter did not indicate pronounced batch effects (Figure 1A,B), as the first principal components showed separation based on immunosuppressive drug use for both LTR and KTR data (Figure 1C,D).

### 3.2. Mycophenolate Versus Mycophenolate Mofetil

We selected mycophenolate (MPA) use as the initial example to evaluate the discovery and differentiation potential of untargeted SWATH mass spectrometry-based profiling workflows. This drug is a cornerstone of immunosuppressive drug treatments aimed at reducing rejection rates after solid organ transplantation [25]. In the mid-1990s, it was initially marketed as mycophenolate mofetil (MMF), a morpholinoethyl ester prodrug with good bioavailability. Later, it became available as an enteric-coated (EC) tablet at higher costs but with reduced incidence of upper gastrointestinal adverse effects [26,27]. As with EC-MPA, the drug is absorbed as MPA in case of the prodrug since “after oral administration, MMF can hardly be detected at any time in plasma because it is rapidly de-esterified in the stomach to produce MPA”, according to the recently published consensus report by the International Association of Therapeutic Drug Monitoring and Clinical Toxicology [25]. Comparable urinary metabolite profiles are thus expected in users of the two products, although possible biotransformation products of intact MMF [28] and/or the cleaved mofetil group [29] might be detected in urine.

**Figure 2:**
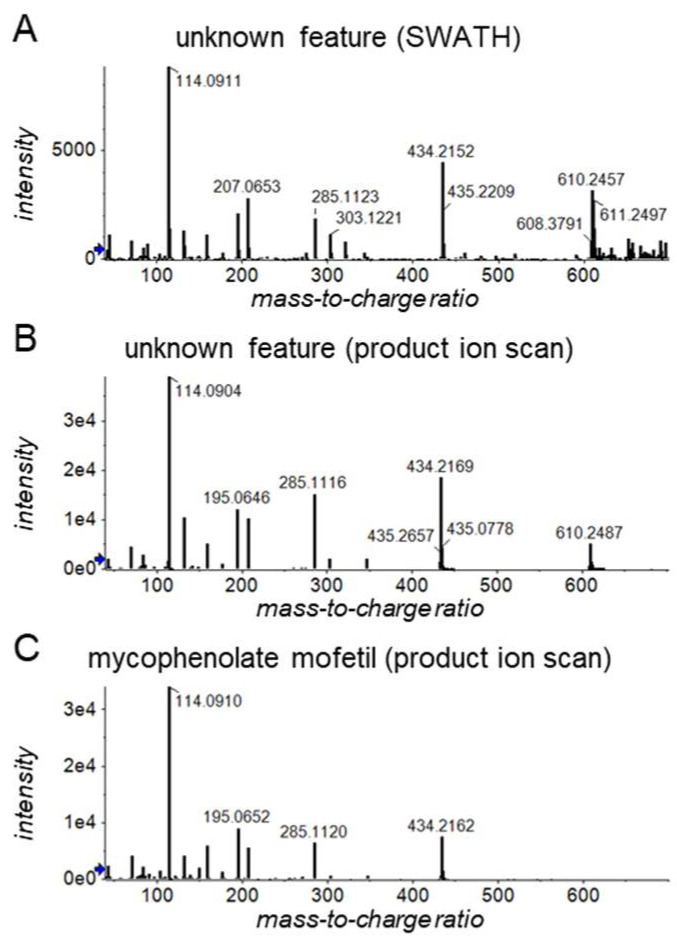
Exemplary (**A**) SWATH and (**B**) product ion scan fragment spectra of an unknown feature (*m*/*z* 610/8.4 min). This feature clusters around mycophenolate-related features in principal component analysis (see Figure 1E,F) and is observed in most but not all mycophenolate-positive samples. (**C**) Product ion scan fragment spectrum of mycophenolate mofetil obtained from a crushed mycophenolate mofetil tablet from the company Sandoz. The blue arrows on the y-axes indicate thresholds for presenting *m*/*z* values.

Unsupervised PCA was initially performed following the observation reported in Section 3.1 that the first principal components showed separation based on immunosuppressive drug use (see Figure 1C,D). The corresponding loading plots (see Figure 1E,F) indicated that strong contributors to PC2 include MPA-related features, such as the ammonium adduct of MPA glucuronide (*m*/*z* 514/9.3 min) and its deglucuronidated form (*m*/*z* 321/9.3 min) that is presumably formed during ion transfer to the mass spectrometer. We also found an unknown feature (*m*/*z* 610/8.4 min) among the five strongest contributors to PC2. Upon inspection of feature intensities across the different samples, this feature was observed in all but one of the MPA-positive LTR and in 80% of the MPA-positive KTR. We extracted the SWATH fragment spectrum of this feature (Figure 2A) and also selected a representative sample, which we reanalyzed to acquire a somewhat cleaner product ion spectrum (Figure 2B). Both spectra suggested the presence of a glucuronidated form of MMF based on the *m*/*z* difference of 176 between the *m*/*z* 610 and 434 peaks, which is indicative of glucuronic acid moieties [30], and the closeness of the *m*/*z* of the 434 peak (i.e., 434.216 Da) to the mass of protonated MMF (i.e., 434.217 Da).

For confirmation, we would have preferred to verify the identity of this compound using a chemical reference standard of MMF glucuronide, but an appropriate standard was unavailable. We could, however, generate a fragment spectrum of the prodrug MMF (Figure 2C), which was highly similar to that of the unknown chemical and essentially only lacked the +176 glucuronide peak. From this, we are confident that the unknown feature represents MMF glucuronide and that it can be useful to differentiate MMF and EC-MPA users.

**Figure 3:**
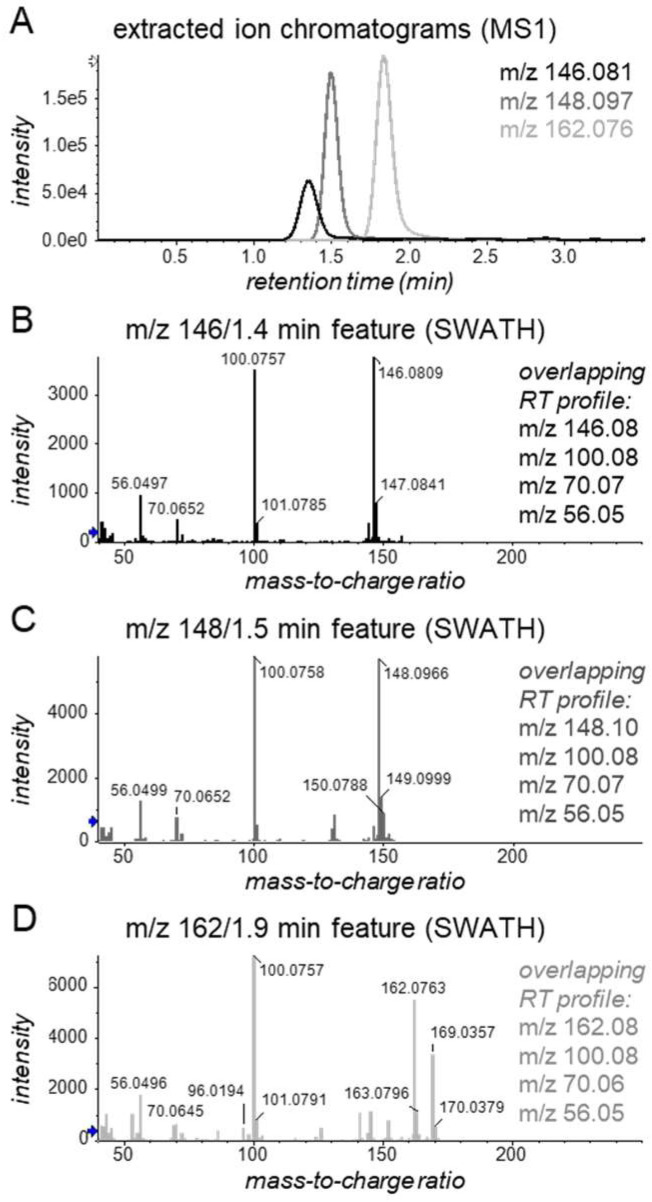
Exemplary (**A**) MS1 extracted ion chromatograms and (**B**–**D**) SWATH fragment spectra of three possible biotransformation products of MMF’s mofetil moiety, which show spectral similarities to each other (see also Figure S4). The first feature (**B**) may represent the previously predicted [29] metabolite N-(2-carboxymethyl)-morpholine, the second (**C**) may represent the previously-predicted [29] metabolite N-(2-hydroxyethyl)-morpholine N-oxide, and the third (**D**) may represent N-(2-carboxymethyl)-morpholine N-oxide, which has not been described previously. The blue and white arrows on the y-axes indicate thresholds for presenting *m*/*z* values.

Next, we performed a univariate t-test on MS1 feature data of the KTR samples aiming to find other differentially abundant features between MMF and EC-MPA users. As expected, this put forward the *m*/*z* 610/8.4 min. feature, a possible deglucuronidated form of this feature, and their isotope peaks among features with the lowest observed *p*-values. Also, among the top 10 most significant features, we found three early-eluting features having nominal *m*/*z* values of 146, 148, and 162 (Figure 3). These features likely represent oxidation products of MMF’s mofetil moiety, but definitive confirmation of their identity would necessitate custom synthesis of candidate structures and further analytical investigation.

Finally, MMF and EC-MPA are often considered as therapeutically-equivalent in clinical studies, despite differences in side effects which triggered the development of EC-MPA [26], and despite differences in metabolite patterns which were found in this study. These differences may provide incentives for reassessing their supposed equivalence, and untargeted SWATH mass spectrometry-based profiling could be a useful technique in this regard. This technique’s differentiation potential is likely applicable to other closely related exposures, although we acknowledge this depends on several factors, for example those related to the structures and abundances of the chemicals of interest.

### 3.3. Cocaine Adulteration

To further evaluate the discovery and differentiation potential of untargeted SWATH mass spectrometry-based profiling workflows, we studied exposure to cocaine and possible adulterants in LTR and KTR. Cocaine abuse has frequently been linked to liver and kidney failure [31], and we expected to encounter this drug in some of the subjects as it is highly addictive and has an estimated lifetime use of around 5% in the Netherlands [32]. We furthermore expected to encounter drug adulterants in the urine of possible cocaine users given that it is rarely provided as pure product [33]. Moreover, we attempted to consider possible temporal trends in cocaine adulteration [34]. Accordingly, we explored publicly available data from a previous study on KTR and (potential) living kidney donors whose urine samples were analyzed with a profiling workflow that is nearly identical to the one used in this study [19].

**Figure 4:**
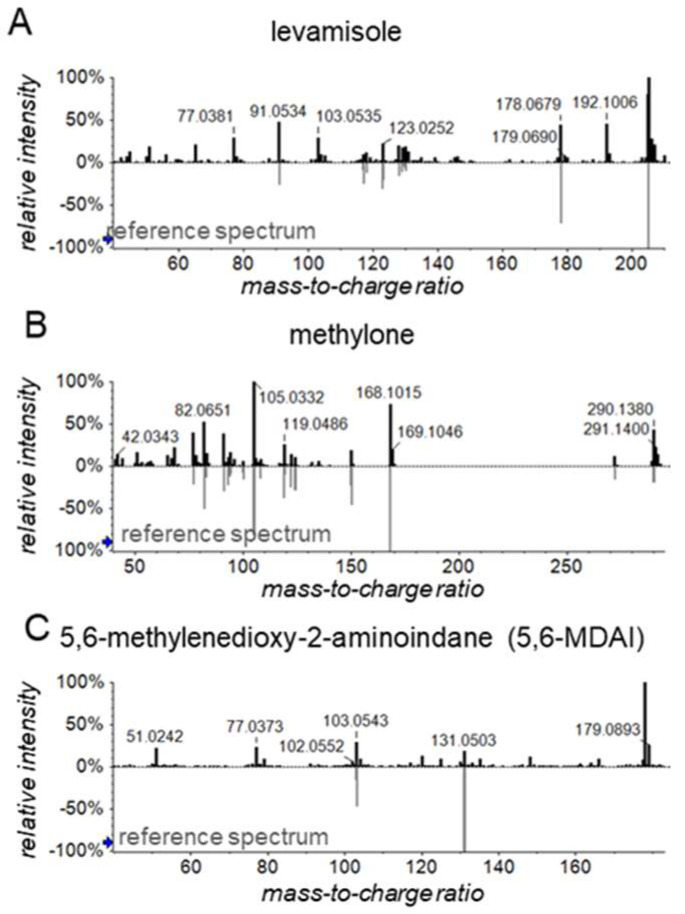
Exemplary spectral library matching results for the possible adulterants (**A**) levamisole, (**B**) methylone, and (**C**) 5,6-methylenedioxy-2-aminoindane (5,6-MDAI), as observed in one of the cocaine-positive samples. See Figures S6–S8 for a more detailed overview of SLM results. The blue arrows on the y-axes indicate thresholds for presenting *m*/*z* values.

Exposure to cocaine was determined by spectral library matching targeting the cocaine-specific metabolite benzoylecgonine in urine samples. This search yielded zero and five positive identifications in LTR and KTR, respectively, and two positive identifications in the previous study on KTR (see Figure S5 and Table S6). The profiling data of benzoylecgonine-positive samples were subsequently subjected to SLM using a commercial forensic spectral library to identify possible cocaine adulterants. This indicated the presence of several therapeutic drugs (e.g., levetiracetam, losartan, metoprolol (5×), oxazepam (2×), ranitidine (2×), sulfamethoxazole, temazepam, trimethoprim, xylometazoline). Also, we found the withdrawn anthelmintic drug levamisole in the two samples from the previous study and the synthetic ecstasy analogs methylone and 5,6-methylenedioxy-2-aminoindane (5,6-MDAI) in one of the samples from the previous study (Figure 4).

With respect to the levamisole identifications, it is interesting that we observed this chemical in both samples from the previous study and none from the present study, considering that the samples were collected around 2009 and 2018, respectively. The identifications in the older samples are consistent with findings from a large-scale study on cocaine adulteration performed in several European countries [35]. This study proposed levamisole as the most commonly used adulterant, being found in more than 50% of the tested cocaine samples in the Netherlands between 2009 and 2013. 

Regarding the methylone and 5,6-MDAI identifications, ecstasy analogs have previously been identified in cocaine preparations. However, these so-called new psychoactive substances (NPS) are frequently encountered as adulterants on the ecstasy market [35,36] but are also used recreationally [37]. In fact, many NPSs are known as ‘legal highs’ for considerable periods of time, since they must be identified by legislative bodies before they can get banned, and this can be many years after market introduction due to a lack of generic legislation for NPS [38]. 

Finally, it is impossible to draw concrete conclusions on cocaine adulteration based on our findings, mostly due to low statistical power, large variability in drug adulteration, and because some users take different illicit drugs at the same time. Nonetheless, our data emphasize the relevance of identifying (pharmacologically active) co-exposures and could be useful for detecting risky lifestyle habits, representing an important but difficult subject to study. Furthermore, this example of drug adulteration underlines the discovery potential of untargeted SWATH mass spectrometry-based profiling workflows, as is particularly illustrated by the 5,6-MDAI findings. This chemical was identified by SLM, and upon further inspection of SLM results, it was found that there were actually two closely eluting signals which could be matched to the 5,6-MDAI reference spectrum (see Figure S8). Using our SWATH data-independent acquisition workflow, we were able to generate informative fragment spectra for both signals, which may correspond to 5,6-MDAI and its positional isomer 4,5-MDAI that are known to produce similar fragment spectra [39]. In fact, SWATH workflows can yield MS2-level information for theoretically all (ionizable) compounds, unlike the more common data-dependent acquisition and MS1-only workflows. SWATH workflows thus yield a ‘digital archive’ for every sample which can be interrogated retrospectively [33] and is particularly interesting for exposures that are not yet ‘on the radar’ when samples are analyzed.

### 3.4. Omeprazole Versus Esomeprazole

Both the MPA example and the cocaine adulteration example presented above addressed differentiation between related exposures based on molecules with different molecular masses. In this example, we focused on more chemically similar exposures, namely the racemic drug omeprazole (i.e., equal mixture of R- and S-omeprazole) and the enantiopure drug esomeprazole (i.e., S-omeprazole), which was developed in response to the considerable interindividual variability in bioavailability, effect, and safety observed among omeprazole users [40,41].

Previous studies (utilizing human liver microsomes) showed stereoselective metabolism of omeprazole, notably demonstrating different patterns of oxidation products for R- and S-omeprazole [42]. Hence, we aimed to explore possible differences in (es)omeprazole metabolism in a real-world clinical setting, for which we first identified (es)omeprazole-positive subjects using information on drug use listed in the available clinical database and molecular evidence of omeprazole exposure. As shown in Table 1, both data sources showed good concordance (90–95%), and the information was combined to extract double-positive and double-negative subjects, for whom it is plausible that they were or were not recently exposed to (es)omeprazole. Exposed subjects were furthermore grouped as possible omeprazole users or possible esomeprazole users based on corresponding clinical database entries.

**Figure 5:**
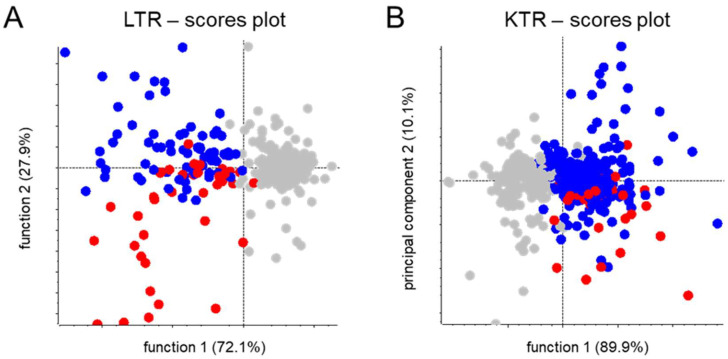
Pareto-scaled scores plots for supervised principal component analysis-discriminant analysis of MS1-level feature data of (es)omeprazole-negative and -positive stable (**A**) liver and (**B**) kidney transplant recipients. Groups were made according to the absence of (es)omeprazole (in gray) and the suspected presence of omeprazole (in blue) and esomeprazole (in red), as was based on combined clinical database- and metabolomics-derived drug use information.

Next, feature-based analyses were performed to find features associated with (es)omeprazole use. This included principal component analysis-discriminant analysis (Figure 5) to yield a global overview of discriminative features and subsequent univariate *t*-test analysis (Table 2) to extract the most discriminating ones. With respect to the latter, it was expected that some features reflect multiple signals, as was also the case for the two MDAI signals which were detected as one single feature due to the closeness of their respective retention times. We thus used the features’ *m*/*z* values and obtained extracted ion chromatograms and the corresponding fragment spectra from raw MS data (see Figures S9–S26). This gave a more reliable overview of the total number of possible (es)omeprazole metabolites in the data, which are listed in the right column of Table 2.

**Figure 6:**
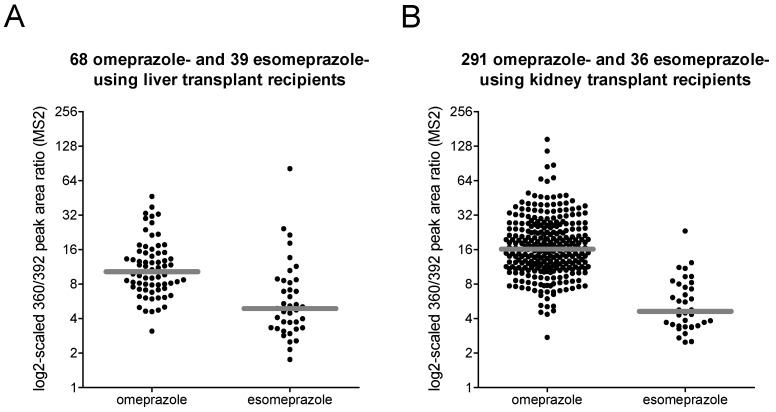
MS2 peak area ratios of the possible (es)omeprazole metabolites *m*/*z* 360 (8.3 min) and *m*/*z* 392 (8.5 min) in (**A**) liver and (**B**) kidney transplant recipients (as black dots). Median values are indicated with a gray line.

In order to find metabolites that discriminate between omeprazole and esomeprazole users, we focused on possible phase I metabolites of (es)omeprazole and calculated peak area ratios following targeted extraction of MS1-level signals. In total, we targeted 21 analytes resulting in 210 ratios which were compared among omeprazole and esomeprazole users (see Table S7). For the compounds showing the most and strongest differences, we also extracted more selective MS2-level signals and compared the corresponding peak area ratios among users of the two drugs (Table 3). Statistical analyses indicated that there are multiple ratios showing pronounced differences between the two groups, although not even the most discriminative ratio allowed for an overlap-free differentiation (see Figure 6).

Finally, none of the ratios showed complete differentiation between omeprazole and esomeprazole users, and it is possible that the study setting, the study samples and/or the selected analytical strategy do not allow for such differentiation. It should, however, also be taken into account that there may be incorrect database entries which could hamper any complete differentiation [17]. Furthermore, there may be external (e.g., co-exposures) and/or genetic factors (e.g., cytochrome p450 polymorphisms) affecting drug metabolism in individual subjects which can have profound influences on drug metabolite patterns [41,43]. In fact, a recent study on 316 (es)citalopram users originating from the same geographical area reported that 80% of the participants were CYP3A4 normal metabolizers and only 56% were CYP2C19 normal metabolizers [44]. Considerable numbers of subjects may thus show altered (es)omeprazole metabolism since CYP2C19 and CYP3A4 are the main enzymes responsible for metabolism of both drugs [41,42]. Accordingly, it would be interesting for future studies to combine our ‘pharmacometabolomics’ data with pharmacogenomics data, for example when attempting to (further) personalize pharmacotherapeutic treatments.

## 4. Conclusions

Untargeted mass spectrometry-based profiling workflows can contribute to increasing data reliability in clinical exposure research by verifying the presence of chemical exposures in biological samples like blood and urine, as underlined by the urinary presence of (es)omeprazole metabolites, which was in good concordance (90–95%) with clinical database-derived (es)omeprazole use. These workflows also have a rather high discovery potential and thus may yield complementary insights into exposures, notably by identifying previously unknown exposure metabolites as we showed for mycophenolate use, by identifying varying combinations of co-exposures as we showed for cocaine use, and by detecting differential abundances of known and previously unknown exposure metabolites as we showed for (es)omeprazole use. Based on these capabilities, profiling methods such as SWATH metabolomics hold considerable potential for differentiating between closely related exposures, which is a major challenge in clinical exposure research. However, this differentiation potential could be further increased by integrating profiling data with, for example, clinical patient characteristics and pharmacogenomics data, as would be desirable given the complexity and the uncertainties inherently associated with exposure research.

## Figures and Tables

**Table 1:** Concordance between clinical database-derived information on (es)omeprazole use and molecular evidence of omeprazole exposure, as is based on urinary signals of five possible oxidation products of omeprazole.

	Clinical Database-Derived Drug Use			
	Non-User	Omeprazole	Esomeprazole	Total
Liver transplant recipients:	196 (62%)	77 (24%)	43 (14%)	316
0 metabolite signals	184 ^1^	7	3	194 (61%)
1–2 metabolite signals	2	2	1	5 (2%)
≥3 metabolite signals	10	68 ^2^	39 ^2^	117 (37%)
Kidney transplant recipients:	214 (38%)	316 (56%)	39 (7%)	569
0 metabolite signals	198 ^1^	16	2	216 (38%)
1–2 metabolite signals	3	9	1	13 (2%)
≥3 metabolite signals	13	291 ^2^	36 ^2^	340 (60%)

^1^ Expected and confirmed (es)omeprazole-negative subjects (“double-negative”). ^2^ Expected and confirmed (es)omeprazole-positive subjects (“double-positive”).

**Table 2:** Overview of nominal *m*/*z* values and possible identities of features which showed strong differentiation between (es)omeprazole-positive and -negative subjects following *t*-test analysis.

*m*/*z*	Possible Biotransformation(s)	Possible Signals
268	various (cysteine metabolite)	1
310	various (mercapturate metabolite)	1
316	dehydroxylation (−16), demethylation (−14)	2
330	dehydroxylation (−16)	1
332	demethylation (−14)	2
346	none (omeprazole)	not detected
360	dehydroxylation (−16), carboxylation (+30)	2
362	hydroxylation (+16)	3
376	carboxylation (+30)	3
378	dihydroxylation (+32)	5
392	hydroxylation (+16), carboxylation (+30)	1
492	dehydroxylation (−16), demethylation (−14), glucuronidation (+176)	4
506	dehydroxylation (−16), glucuronidation (+176)	2
508	demethylation (−14), glucuronidation (+176)	8
522	glucuronidation (+176)	7
536	dehydroxylation (−16), carboxylation (+30), glucuronidation (+176)	1
538	hydroxylation (+16), glucuronidation (+176)	5
552	carboxylation (+30), glucuronidation (+176)	1
554	dihydroxylation (+32), glucuronidation (+176)	2

**Table 3:** Mann Whitney U *p*-value ^1^ matrix for differences in MS2-level ^2^ ratios of possible phase I metabolites of omeprazole when comparing omeprazole and esomeprazole users ^3^ among liver transplant recipients (LTR) and kidney transplant recipients (KTR).

*m*/*z*		332	360	362	376	378	392	
	RT (min)	7.9	8.3	10.3	7.2	7.7	8.5	
332	7.9		1.4 × 10^−4^	1.2 × 10^−8^	0.18	1.4 × 10^−4^	3.9 × 10^−10^	KTR
360	8.3	0.54		4.7 × 10^−11^	0.03	0.06	2.7 × 10^−17^	
362	10.3	1.8 × 10^−4^	6.3 × 10^−5^		1.5 × 10^−9^	1.9 × 10^−12^	0.05	
376	7.2	0.95	0.61	2.2 × 10^−4^		0.013	2.8 × 10^−15^	
378	7.7	3.2 × 10^−3^	3.2 × 10^−3^	3.8 × 10^−6^	0.01		4.5 × 10^−11^	
392	8.5	3.5 × 10^−6^	8.1 × 10^−7^	0.06	6.2 × 10^−6^	1.2 × 10^−6^		
		LTR						

^1^ Significance testing was based on an alpha of 0.05 and Bonferroni correction. Statistically significant associations are presented in scientific format. ^2^ Ratios were derived from the following SRM-like traces: [324–339] → 165.0117 (332), [352–367] → 149.0709 (360), [352–367] → 298.1550 (362), [366–381] → 149.0709 (376), [366–381] → 212.0376 (378), [380–395] → 149.0709 (392). ^3^ Only “double-positive” subjects were included, and subjects were assigned as omeprazole or esomeprazole user based on information present in the clinical database.

## Data Availability

The metabolomics datasets generated in this study can be found at: https://doi.org/10.26037/yareta:64ruex2sxff5nenyfyexurzs3m (as sub-studies 2 and 3).

## Supplementary Materials

The following supporting information can be downloaded at: https://www.mdpi.com/article/10.3390/metabo12100942/s1, Table S1: Overview of internal standards; Table S2: Overview of LC-MS analytical parameters; Table S3: Overview of PeakView chemical identification settings; Table S4: Overview of MarkerView data (pre)processing settings; Table S5: Baseline characteristics of the stable liver (LKR) and kidney transplant recipients (KTR) included in this study; Table S6: Quantitative data for cocaine and selected cocaine metabolites in the urine of samples in which benzoylecgonine was identified; Table S7: *p*-value matrix for differences in MS1-level ratios of possible phase I metabolites of omeprazole when comparing omeprazole and esomeprazole users among liver transplant recipients and kidney transplant recipients; Figures S1 and S2: Exemplary extracted ion chromatograms and fragment spectra of five possible oxidation products of omeprazole; Figure S3: Extracted ion chromatograms and fragment spectra of 5-hydroxyomeprazole-D_3_, omeprazole sulfone, and omeprazole; Figure S4: Exemplary extracted ion chromatograms and fragment spectra of three possible biotransformation products of MMF’s mofetil moiety, and an extracted ion chromatogram and fragment spectrum of N-(2-hydroxyethyl)-morpholine. Figure S5: Extracted ion chromatogram and fragment spectrum of benzoylecgonine and spectral library matching results of benzoylecgonine-positive samples; Figure S6: Extracted ion chromatogram and fragment spectrum of levamisole and spectral library matching results of levamisole-positive samples; Figure S7: Extracted ion chromatogram and fragment spectrum of methylone and spectral library matching result of the methylone-positive sample; Figure S8: Extracted ion chromatogram and fragment spectra of two possible methylenedioxy-2-aminoindanes (MDAI) as well as spectral library matching result for 5,6-MDAI, extracted ion chromatograms of presumed MDAI-derived fragments, and structural formulas of 5,6-MDAI and 4,5-MDAI, as observed in the 5,6-MDAI-positive sample; Figures S9–S26: Exemplary extracted ion chromatogram and fragment spectrum of a possible phase I and II metabolites of omeprazole.
