# Supplementary material for: Assessing the Potential of Untargeted SWATH Mass Spectrometry-Based Metabolomics to Differentiate Closely Related Exposures in Observational Studies
Source: Metabolites. 2022 Oct 4;12(10):942. doi: 10.3390/metabo12100942 (PMC9611019; doi:10.3390/metabo12100942)
Supplement: Supplementary file 1 [file metabolites-12-00942-s001.zip › metabolites-1923385-supplementary.pdf]

metabolites

MDPI

# Supplementary Material

**Supplemental Table S1.** Overview of internal standards.

| Chemical | Isotope label | Supplier | Catalogue number |
|---|---|---|---|
| Acetaminophen | ring-$D_4$ | Toronto Research Chemicals | A161222 |
| Diclofenac | ring-$^{13}C_6$ | Sigma-Aldrich | 35361 |
| Naproxen | methoxy-$D_3$ | Sigma-Aldrich | 32104 |
| Caffeine | trimethyl-$^{13}C_3$ | Sigma-Aldrich | C-082 |
| Cotinine | N-methyl-$D_3$ | Sigma-Aldrich | C-035 |

**Supplemental Table S2.** Overview of LC-MS analytical parameters.

| Setting | Value |
|---|---|
| *Analytical setup:* | |
| LC pump | Dionex Ultimate 3000 RS Binary Pump (Cat. No. HPG-3400RS) |
| LC autosampler | Dionex Ultimate 3000 TS Autosampler (Cat. No. WPS-3000TRS) |
| LC column oven | Dionex Ultimate 3000 RS Column Compartment (Cat. No. TCC-3000RS) |
| LC operation software | Dionex Chromeleon Software (version 6.80 SR10, Build 2818) |
| MS instrument | SCIEX TripleTOF® 5600 Mass Spectrometer (Cat. No. 1032150) |
| MS calibration device | SCIEX Calibrant Delivery System (Cat. No. ISG-002019) |
| MS operation software | SCIEX Analyst TF Software (version 1.7.1, Build 1163) |
| *LC parameters:* | |
| Autosampler temperature | 6 °C |
| Column temperature | 40 °C |
| Analytical column | Dr. Maisch ReproSil Gold 120, 2.5 µm, 120 Å, 2.0 × 150 mm (Cat. No. r125.9g.s1502) |
| Trapping column | Dr. Maisch ReproSil-Pur 120 C18-AQ, 2.4 µm, 120 Å, 2.0 × 5 mm (Cat. No. r124.aq.v0002) |
| Mobile phase A | 5 mM ammonium formate (Fluka; Cat. No. 17843) and 0.1% formic acid (Merck; Cat. No. 1.00264.0100) in water (Chemsolute; Cat. No. 470.2500) |
| Mobile phase B | Methanol (Fisher Science; Cat. No. M/4058/17) |
| Trapping settings | 0.3 mL/min at 2% B |
| Flow rate | 0.3 mL/min |
| LC program | 0.00 min: 5% B<br>0.20 min: column switch<br>0.50 min: 5% B<br>12.50 min: 80% B<br>14.00 min: 100% B<br>17.00 min: 100% B<br>17.01 min: 5% B<br>19.00 min: 5% B |
| *MS parameter:* | |
| Source temperature (TEM) | 500 °C |
| Curtain gas (CUR) | 35 psi |
| Nebulizer gas (GS1) | 40 psi |
| Heater gas (GS2) | 40 psi |
| IonSpray voltage floating (ISVF) | 5,200 V |
| Declustering potential (DP) | 80 V |
| Collision energy (CE) MS1 | 10 V |
| Collision energy (CE) MS2 | 40 V |
| Collision energy spread (CES) MS1 | 0 V |
| Collision energy spread (CES) MS2 | 30 V |
| Accumulation time MS1 | 50 ms |
| Accumulation time MS2 | 23 ms |
| TOF MS1 range | m/z 100-1,250 |
| TOF MS2 range | m/z 40-1,250 |
| SWATH precursor isolation windows | Experiment 1: m/z 100-115<br>Experiment 2: m/z 114-129<br>Experiment 3: m/z 128-143<br>Experiment 4: m/z 142-157<br>Experiment 5: m/z 156-171 |

| | |
|---|---|
| | Experiment 6: m/z 170-185 |
| | Experiment 7: m/z 184-199 |
| | Experiment 8: m/z 198-213 |
| | Experiment 9: m/z 212-227 |
| | Experiment 10: m/z 226-241 |
| | Experiment 11: m/z 240-255 |
| | Experiment 12: m/z 254-269 |
| | Experiment 13: m/z 268-283 |
| | Experiment 14: m/z 282-297 |
| | Experiment 15: m/z 296-311 |
| | Experiment 16: m/z 310-325 |
| | Experiment 17: m/z 324-339 |
| | Experiment 18: m/z 338-353 |
| | Experiment 19: m/z 352-367 |
| | Experiment 20: m/z 366-381 |
| | Experiment 21: m/z 380-395 |
| | Experiment 22: m/z 394-409 |
| | Experiment 23: m/z 408-423 |
| | Experiment 24: m/z 422-437 |
| | Experiment 25: m/z 436-451 |
| | Experiment 26: m/z 450-465 |
| | Experiment 27: m/z 454-479 |
| | Experiment 28: m/z 478-493 |
| | Experiment 29: m/z 492-507 |
| | Experiment 30: m/z 506-521 |
| | Experiment 31: m/z 520-535 |
| | Experiment 32: m/z 534-549 |
| | Experiment 33: m/z 548-563 |
| | Experiment 34: m/z 562-577 |
| | Experiment 35: m/z 576-591 |
| | Experiment 36: m/z 590-605 |
| | Experiment 37: m/z 604-900 |
| | Experiment 38: m/z 899-1,250 |
| Total cycle time | ± 1.0 s |

**Supplemental Table S3.** Overview of PeakView chemical identification settings.

| Setting | Value |
|---|---|
| *Calculations:* | |
| Do not calculate details for XIC with intensity < N counts or S:N < M | N = 1000, M = 100 |
| Default XIC width (Da) | 0.01 |
| Default retention time width | 0.2 |
| Default threshold (cps) | 100 |
| Default threshold (ratio of control) | 1 |
| Non-targeted peak finding | Not selected |
| Formula Finder | Not selected |
| *Library searching:* | |
| Algorithm to use during library search | Candidate search |
| Results sorted by | Fit |
| Libraries to search | Sciex Forensics version 1.1<br>Custom spectral library |
| Precursor mass tolerance | 0.4 Da |
| Collision energy tolerance | Not selected |
| Retention time tolerance | Not selected |
| Mass tolerance | 0.4 Da |
| Use polarity | Selected |
| Use collision energy spread | Not selected |
| Use compound specific purity | Not selected |
| Maximum number of hits | 5 |
| Intensity threshold | 0.05 |
| Minimal purity | 10% |
| Intensity factor | 5 |
| *Confidence settings:* | |
| Mass error – hit | < 5 ppm |
| Mass error – potential hit | < 10 ppm |
| Mass error – contribution to combined score | 40% |
| Isotope ratio – hit | < 10% |
| Isotope ratio – potential hit | < 20% |
| Isotope ratio – contribution to combined score | 10% |
| Library score – hit | > 70 |
| Library score – potential hit | > 30 |
| Library score – contribution to combined score | 50% |
| Combined score for a positive identification | ≥ 80% |

**Supplemental Table S4.** Overview of MarkerView data (pre)processing settings.

| Setting | Value |
|---|---|
| *Feature finding:* | |
| Experiment | MS1-only or all MS2 experiments |
| Minimum retention time | 0.50 min |
| Maximum retention time | 16.00 min |
| Subtraction offset | 15 scans |
| Subtraction multiplication factor | 1.3 |
| Noise threshold | 5 |
| Minimum spectral peak width | 5 ppm |
| Minimum retention time peak width | 5 scans |
| Assign charge states | Enabled |
| *Feature alignment:* | |
| Retention time tolerance | 0.50 min |
| Mass tolerance | 0.01 Da |
| *Feature filtering:* | |
| Maximum number of peaks | 8,000,000* |
| Remove peaks in < N samples | Disabled |
| Isotope filtering | Disabled |
| Intensity threshold | 5 |
| Use exclusion list | Disabled |
| Retention time filtering | Disabled |
| Use area integrated from raw data, not from original peak finding | Disabled |
| *MS1-level normalization**:* | |
| Normalization method | Most-Likely Ratio |
| *MS2-level normalization**:* | |
| Normalization feature m/z value (diclofenac-$^{13}C_6$ fragment ion) | 256.04 |
| Normalization feature experiment (diclofenac-$^{13}C_6$ fragment ion) | 16 |
| Normalization feature retention time (diclofenac-$^{13}C_6$ fragment ion) | 13 min |
| *Principle component analysis:* | |
| PCA preprocessing - Weighting | None |
| PCA preprocessing - Scaling | Pareto |
| Perform PCA-DA (supervised) | Disabled |
| *Principle component analysis-discriminant analysis:* | |
| PCA preprocessing - Weighting | None |
| PCA preprocessing - Scaling | Pareto |
| Perform PCA-DA (supervised) | Enabled |
| *T-test:* | |
| Samples per group for “first to last” comparison | Disabled |
| Use Welch t-test | Disabled |

*: This parameter was set high enough to prevent peaks from getting filtered at this stage.
**: Normalization was performed after feature peak list generation.

**Supplemental Table S5.** Baseline characteristics of the stable liver (LKR) and kidney transplant recipients (KTR) included in this study.

| Variable* | LTR | KTR |
|---|---|---|
| Samples, number | 316 | 570 |
| Females, % | 43% | 42% |
| Age, years | 55.0 (14.6) | 55.4 (13.1) |
| Body mass index (BMI), kg/m$^2$ | 26.7 (4.9) | 27.4 (5.0) |
| Body length, cm | 172 (10) | 173 (10) |
| Body weight, kg | 79.6 (17.0) | 81.7 (16.6) |
| Waist circumference, cm | 97.2 (15.8) | 99.6 (14.2) |
| Hip circumference, cm | 102.3 (10.4) | 102.4 (10.3) |
| Serum albumin, g/L | 44.0 (3.4) | 43.3 (2.9) |
| Estimated glomerular filtration rate (eGFR), mL/min/1.73m$^2$ | 74.9 (26.2) | 53.1 (19.2) |

**Abbreviations:** BMI, body mass index; LTR, liver transplant recipient; KTR, kidney transplant recipient.
*: continuous variables are presented as mean (standard deviation).

**Supplemental Table S6.** Quantitative data for cocaine and selected cocaine metabolites in the urine of samples in which benzoylecgonine was identified.

| Sample | Cocaine | Benzoylecgonine | Ecgonine methyl ester | Cocaethylene |
|---|---|---|---|---|
| X-1 | <25 ng/mL | <25 ng/mL | <25 ng/mL | <25 ng/mL |
| X-2 | <25 ng/mL | <25 ng/mL | <25 ng/mL | <25 ng/mL |
| X-3 | <25 ng/mL | <25 ng/mL | <25 ng/mL | <25 ng/mL |
| X-4 | <25 ng/mL | <25 ng/mL | <25 ng/mL | <25 ng/mL |
| X-5 | <25 ng/mL | 1251 ng/mL | 229 ng/mL | <25 ng/mL |
| Y-1 | <25 ng/mL | 1524 ng/mL | 200 ng/mL | <25 ng/mL |
| Y-2 | 1167 ng/mL | 137 ng/mL | 6332 ng/mL | <25 ng/mL |

**Supplemental Table S7.** P-value matrix for differences in MS1-level ratios of possible phase I metabolites of omeprazole when comparing omeprazole and esomeprazole users among liver transplant recipients (in grey) and kidney transplant recipients (in black). With regard to drug use, only "double-positive" subjects were included, and subjects were assigned as omeprazole or esomeprazole user based on information present in the clinical database.

| m/z | | 268 | 310 | 316 | | 330 | 332 | | 360 | | 362 | | | 376 | | | 378 | | | | | 392 |
|---|---|---|---|---|---|---|---|---|---|---|---|---|---|---|---|---|---|---|---|---|---|---|
| | RT | 5.6 | 6.7 | 7.9 | 8.8 | 9.8 | 6.7 | 7.9 | 8.3 | 9.2 | 8.7 | 9.3 | 10.3 | 6.3 | 7.2 | 8.2 | 7.7 | 8.4 | 9.0 | 9.2 | 9.5 | 8.5 |
| 268 | 5.6 | | ns | ns* | ns* | ns* | ns | ns* | $2e^{-8}$ | $2e^{-5}$ | ns | ns* | $1e^{-5}$ | ns | $2e^{-5}$ | ns | $6e^{-8}$ | ns* | $9e^{-6}$ | $3e^{-6}$ | $2e^{-5}$ | $2e^{-4}$ |
| 310 | 6.7 | ns | | ns | ns* | ns | ns | $6e^{-5}$ | $8e^{-12}$ | $2e^{-4}$ | ns | ns* | $6e^{-6}$ | ns | $1e^{-7}$ | ns* | $6e^{-8}$ | ns* | $2e^{-4}$ | $4e^{-5}$ | ns* | ns* |
| 316 | 7.9 | ns | ns | | ns | ns | ns | $5e^{-5}$ | $4e^{-10}$ | ns | ns* | ns | ns | ns | $2e^{-6}$ | ns* | $1e^{-8}$ | ns* | ns | ns* | ns | ns |
| | 8.8 | ns | ns* | ns | | ns | ns | $1e^{-11}$ | $4e^{-17}$ | ns | ns* | ns | ns | ns* | $8e^{-12}$ | ns* | $5e^{-11}$ | $1e^{-6}$ | ns* | ns* | ns | ns |
| 330 | 9.8 | ns | ns | ns | ns | | ns | ns* | $4e^{-8}$ | ns* | ns | ns | ns* | ns | $3e^{-6}$ | ns* | $5e^{-8}$ | ns* | ns | $2e^{-4}$ | ns* | ns |
| 332 | 6.7 | ns | ns | ns | ns | ns | | ns* | $3e^{-6}$ | ns | ns | ns | ns | ns | ns* | ns | $8e^{-5}$ | ns | ns | ns* | ns | ns |
| | 7.9 | ns* | ns* | $2e^{-4}$ | $4e^{-10}$ | ns* | ns* | | $2e^{-6}$ | $7e^{-8}$ | ns | $1e^{-4}$ | $1e^{-7}$ | $6e^{-5}$ | ns* | ns | $4e^{-5}$ | ns | $6e^{-10}$ | $7e^{-7}$ | $2e^{-6}$ | $2e^{-8}$ |
| 360 | 8.3 | $2e^{-4}$ | $1e^{-5}$ | $2e^{-7}$ | $5e^{-12}$ | $2e^{-4}$ | ns* | ns* | | $2e^{-13}$ | ns | $2e^{-8}$ | $1e^{-11}$ | $3e^{-13}$ | ns* | ns | ns | $3e^{-7}$ | $1e^{-13}$ | $1e^{-9}$ | $6e^{-10}$ | $2e^{-18}$ |
| | 9.2 | ns* | ns* | ns | ns | ns | ns | $5e^{-5}$ | $5e^{-7}$ | | ns* | ns | ns | $2e^{-4}$ | $2e^{-12}$ | ns* | $1e^{-12}$ | $1e^{-8}$ | ns | ns* | ns | ns |
| 362 | 8.7 | ns | ns | ns | ns | ns | ns | ns | ns | ns* | | ns* | ns* | ns | ns | ns | ns* | ns | ns* | ns* | ns* | ns* |
| | 9.3 | ns | ns | ns | ns | ns | ns | ns* | $1e^{-4}$ | ns | ns | | ns | ns* | $1e^{-6}$ | ns* | $7e^{-10}$ | $5e^{-5}$ | ns | ns* | $6e^{-5}$ | ns |
| | 10.3 | ns* | $7e^{-6}$ | ns | ns | ns* | ns | $7e^{-6}$ | $8e^{-8}$ | ns | ns* | ns | | ns* | $2e^{-10}$ | ns* | $1e^{-11}$ | $1e^{-7}$ | ns | ns* | ns | ns |
| 376 | 6.3 | ns | ns | ns | ns* | ns | ns | ns* | $2e^{-6}$ | ns* | ns | ns | ns* | | $5e^{-9}$ | ns | $2e^{-8}$ | ns* | ns* | ns* | ns* | $9e^{-5}$ |
| | 7.2 | ns* | ns* | ns* | $8e^{-6}$ | ns* | ns* | ns | ns | $3e^{-7}$ | ns | ns* | $1e^{-6}$ | ns* | | ns | ns* | ns* | $4e^{-11}$ | $1e^{-8}$ | $2e^{-8}$ | $9e^{-16}$ |
| | 8.2 | ns | ns | ns | ns | ns | ns | ns | ns | ns* | ns | ns | ns* | ns | ns | | ns | ns | ns* | $2e^{-4}$ | ns* | ns* |
| 378 | 7.7 | $2e^{-4}$ | $2e^{-4}$ | $5e^{-6}$ | $2e^{-6}$ | $1e^{-4}$ | ns* | ns* | ns | $3e^{-7}$ | ns* | $1e^{-5}$ | $9e^{-8}$ | $1e^{-4}$ | ns* | ns | | $2e^{-6}$ | $2e^{-13}$ | $4e^{-12}$ | $3e^{-11}$ | $1e^{-11}$ |
| | 8.4 | ns* | ns | ns* | $2e^{-4}$ | ns* | ns | ns | ns* | $4e^{-5}$ | ns | ns* | $5e^{-6}$ | ns | ns | ns | ns* | | $1e^{-8}$ | $9e^{-7}$ | $1e^{-6}$ | $3e^{-13}$ |
| | 9.0 | ns | ns | ns | ns | ns | ns | $2e^{-4}$ | $3e^{-6}$ | ns* | ns | ns | ns* | ns | ns* | ns | $7e^{-6}$ | ns* | | ns* | ns | ns |
| | 9.2 | ns* | ns* | ns | ns | ns | ns | ns* | ns* | ns | ns | ns | ns | ns | ns* | ns | $3e^{-5}$ | ns* | ns | | ns | ns* |
| | 9.5 | ns | ns* | ns | ns | ns | ns | ns* | $1e^{-4}$ | ns | ns | ns | ns | ns | ns* | ns | $3e^{-5}$ | ns* | ns | ns | | ns |
| 392 | 8.5 | ns | ns* | ns | ns | ns | ns | $1e^{-5}$ | $6e^{-8}$ | ns* | ns | ns | ns | ns | $8e^{-6}$ | ns | $6e^{-6}$ | $2e^{-6}$ | ns | ns | ns | |

ns: nonsignificant, as was based on Mann-Whitney U nonparametric testing using an alpha of 0.05 and Bonferroni correction. Nonsignificant associations with a P-value between the non-corrected and the Bonferroni-corrected alpha level are indicated with an asterisk (*).

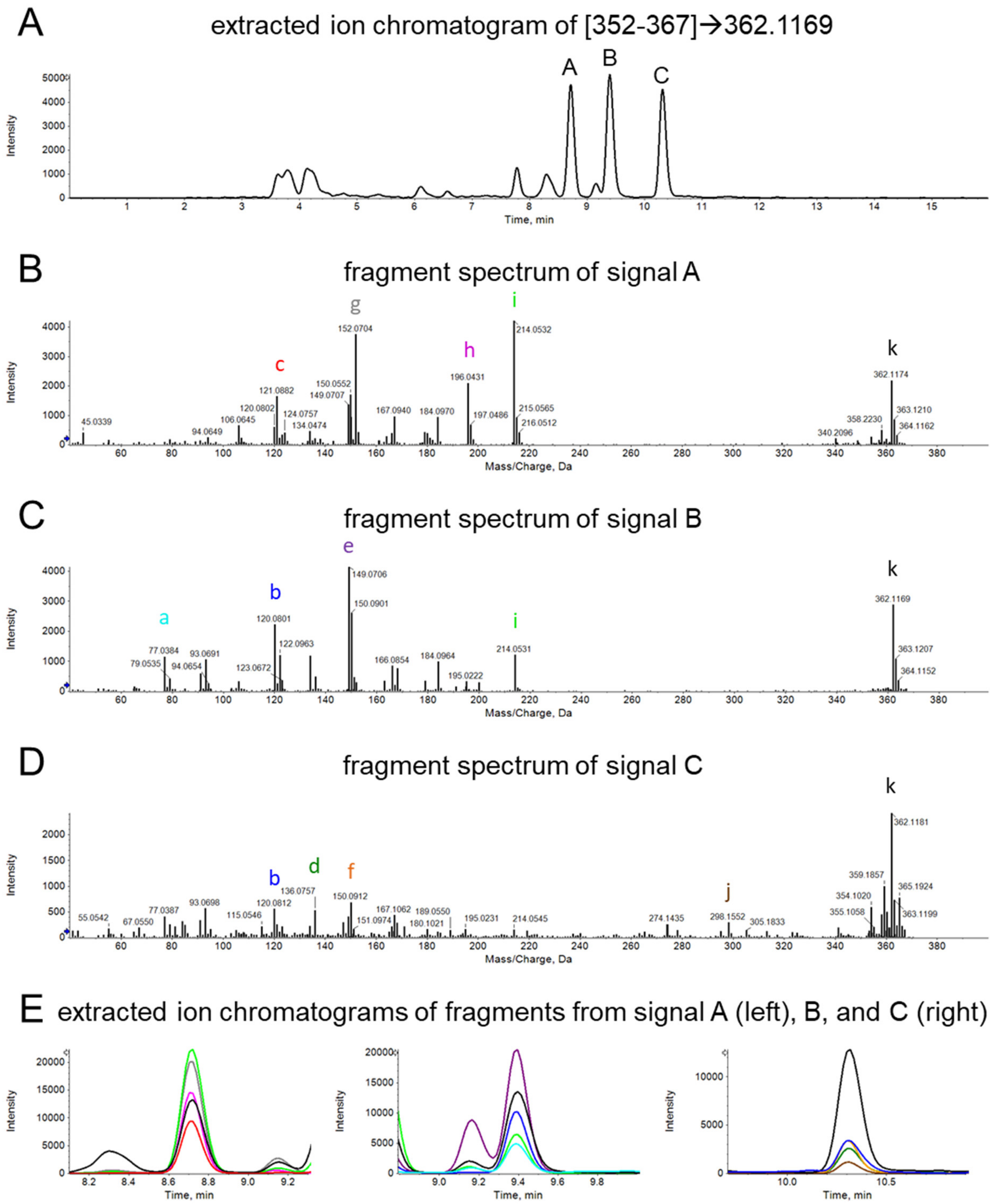


**Supplemental Figure S1.** Exemplary (**A**, **E**) extracted ion chromatograms and (**B**, **C**, **D**) fragment spectra of three possible oxidation products (+16) of omeprazole as observed in one of the omeprazole-positive samples. Quantifier traces for signals A, B, and C are respectively indicated in light green (m/z 214.0532, i), light green (m/z 214.0532, i), and brown (m/z 298.1550, j). The blue and white arrows on the y-axes indicate thresholds for presenting m/z values.

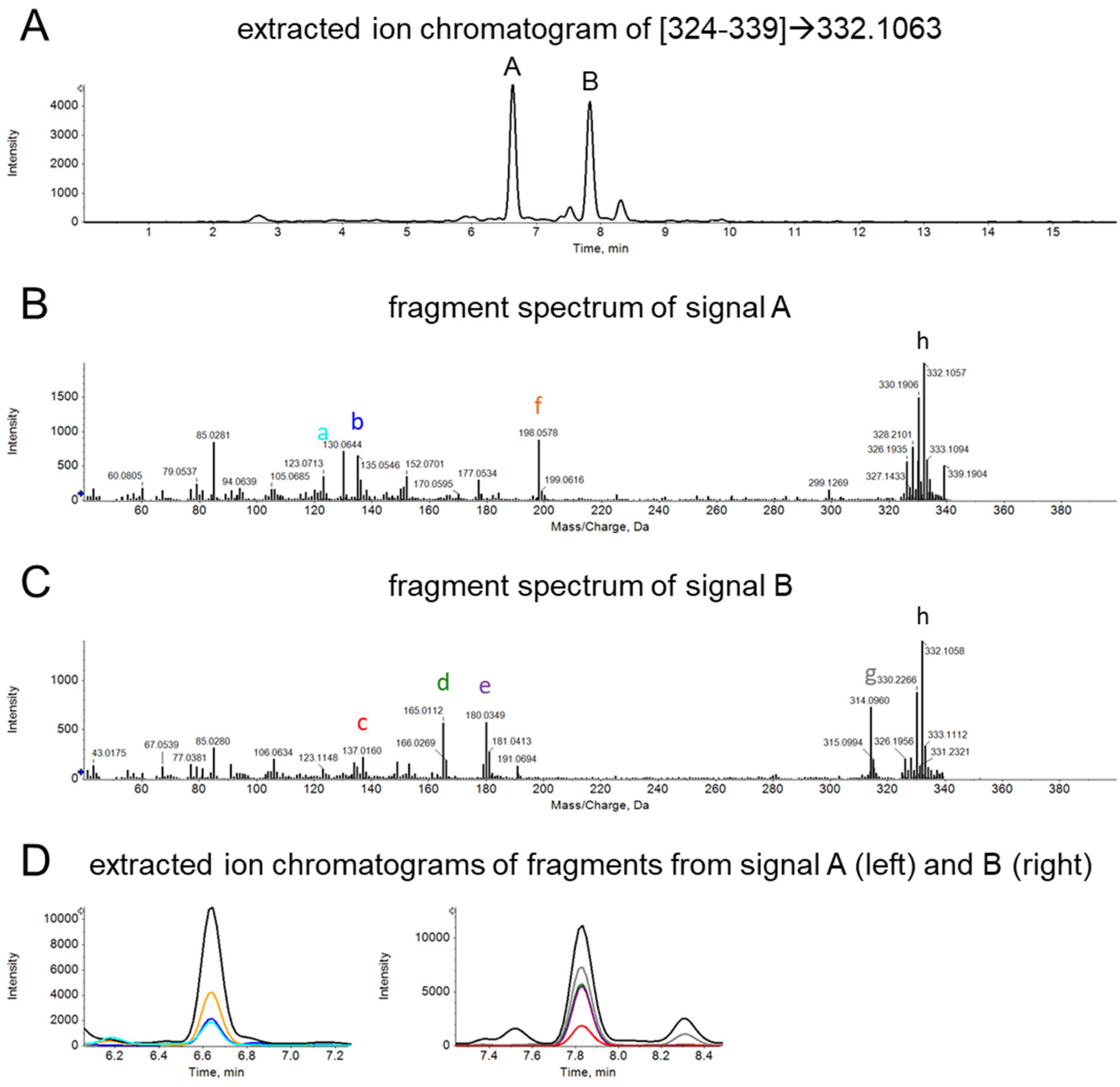


**Supplemental Figure S2.** Exemplary (**A**, **D**) extracted ion chromatograms and (**B**, **C**) fragment spectra of two possible oxidation products (-14) of omeprazole as observed in one of the omeprazole-positive samples. Quantifier traces for signals A and B are respectively indicated in orange (m/z 198.0583, f) and dark green (m/z 165.0117, d). The blue and white arrows on the y-axes indicate thresholds for presenting m/z values.

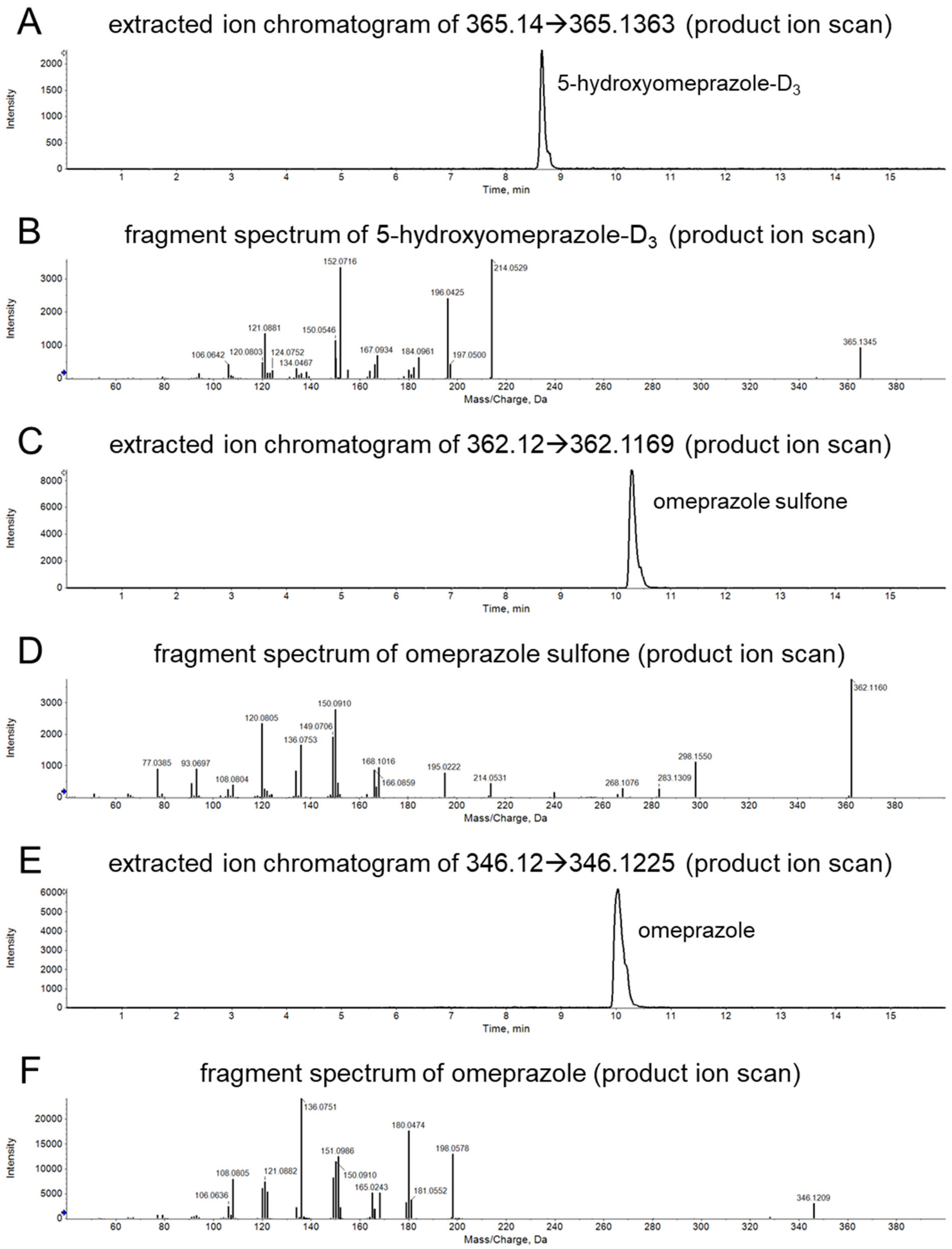


**Supplemental Figure S3**. (**A**, **C**, **E**) Extracted ion chromatograms and (**B**, **D**, **F**) fragment spectra of (**A**, **B**) 5-hydroxyomeprazole-$D_3$ (Toronto Research Chemicals, Cat. No. H948864), (**C**, **D**) omeprazole sulfone (Sigma-Aldrich, Cat. No. O0151000), and (**E**, **F**) omeprazole (crushed granules from a omeprazole capsule from the company Healthypharm). The blue and white arrows on the y-axes indicate thresholds for presenting m/z values.

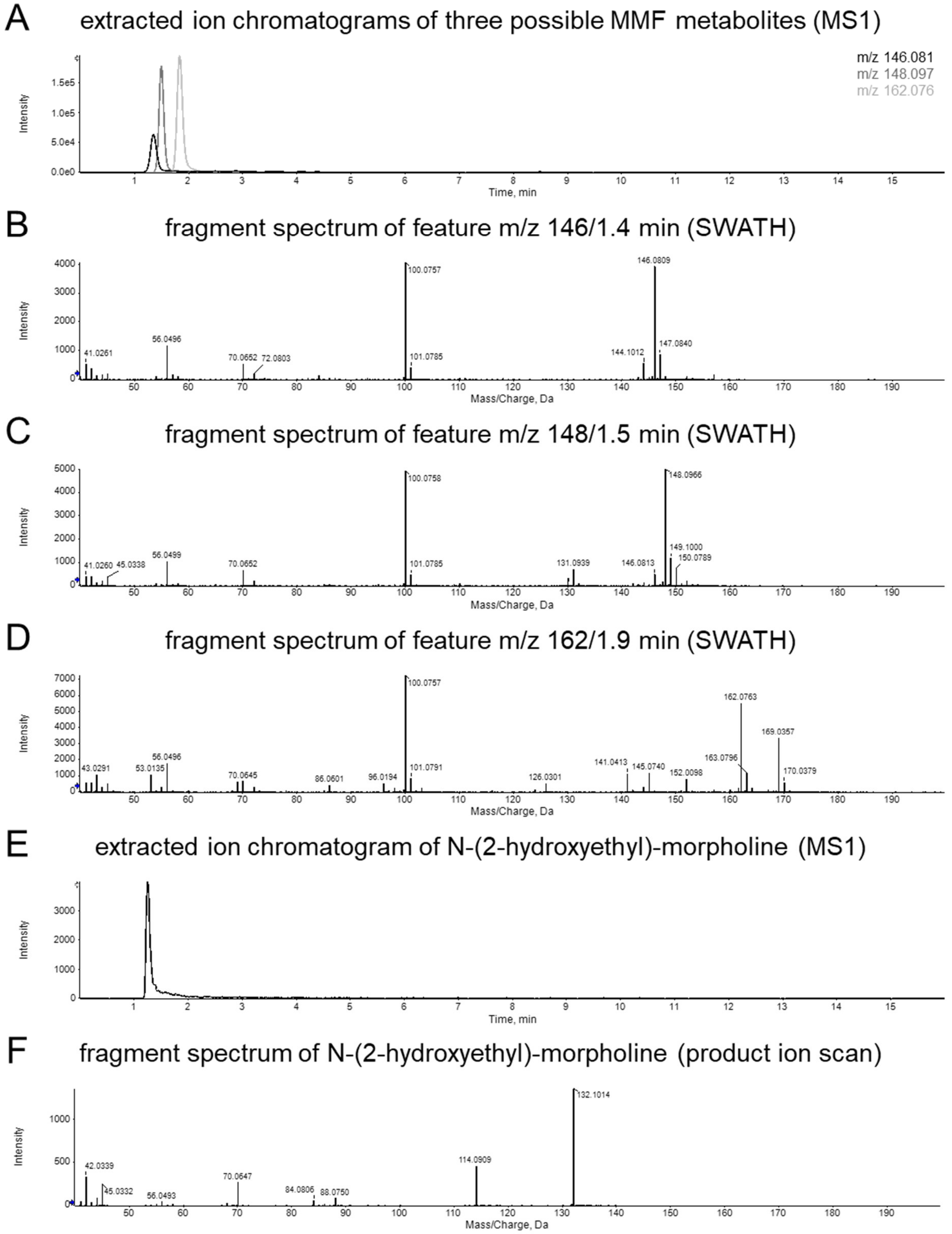


**Supplemental Figure S4**. Exemplary (**A**) extracted ion chromatograms and (**B**, **C**, **D**) fragment spectra of three possible biotransformation products of MMF's mofetil moiety. (**E**) Extracted ion chromatogram and (**F**) fragment spectrum of N-(2-hydroxyethyl)-morpholine (Sigma-Aldrich, Cat. No. H28203), which was not detected but has been described as possible biotransformation product of MMF's mofetil moiety. The blue and white arrows on the y-axes indicate thresholds for presenting m/z values.

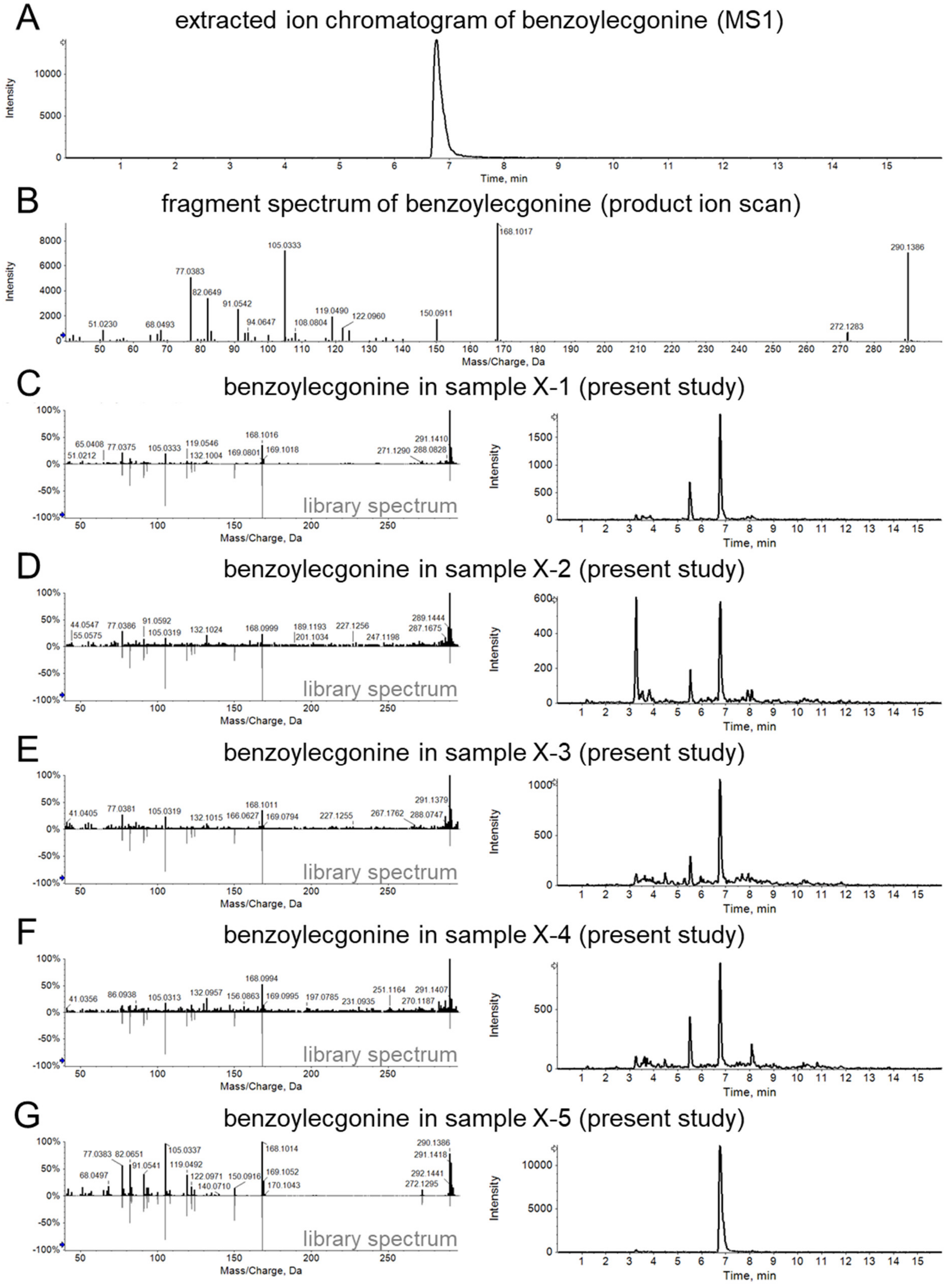


**Supplemental Figure S5.** (**A**) Extracted ion chromatogram and (**B**) fragment spectrum of benzoylecgonine (Lipomed, Cat. No. COC-204-FB) and (**C**-**I**) spectral library matching results of benzoylecgonine-positive samples. The blue and white arrows on the y-axes indicate thresholds for presenting m/z values.

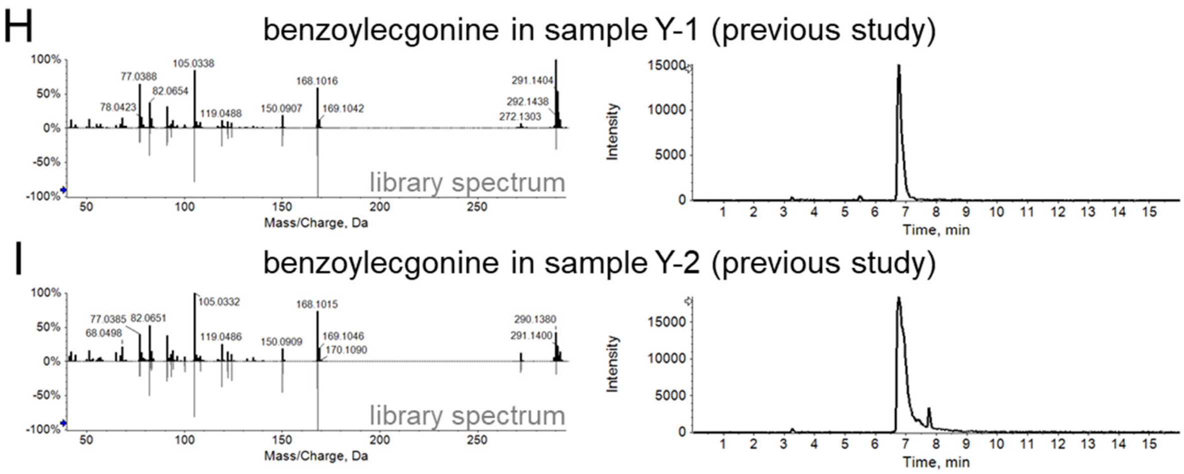


**Supplemental Figure S5.** *Cont*.

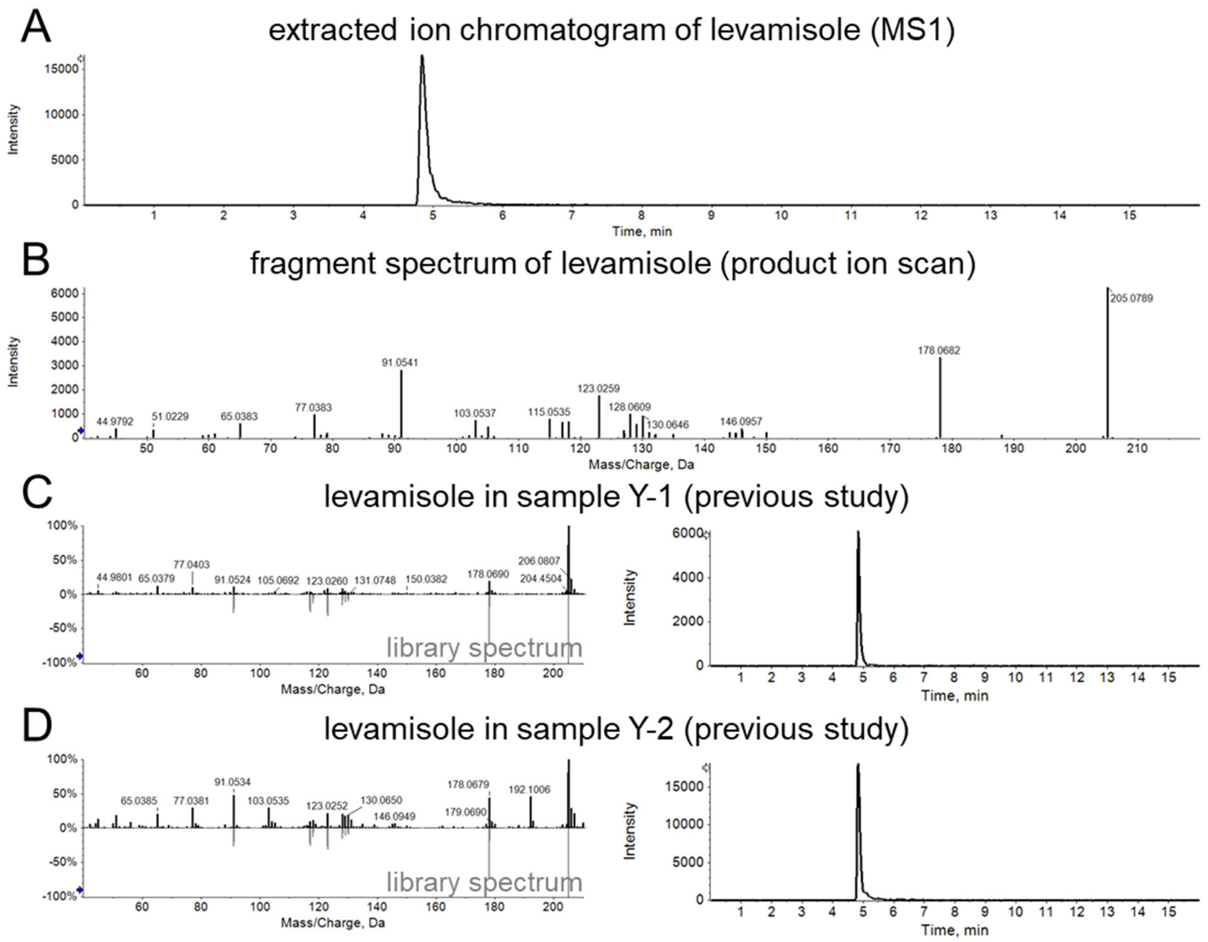


**Supplemental Figure S6.** (**A**) Extracted ion chromatogram and (**B**) fragment spectrum of levamisole (Sigma-Aldrich, Cat. No. L9756) and (**C**-**D**) spectral library matching results of levamisole-positive samples. The blue and white arrows on the y-axes indicate thresholds for presenting m/z values.

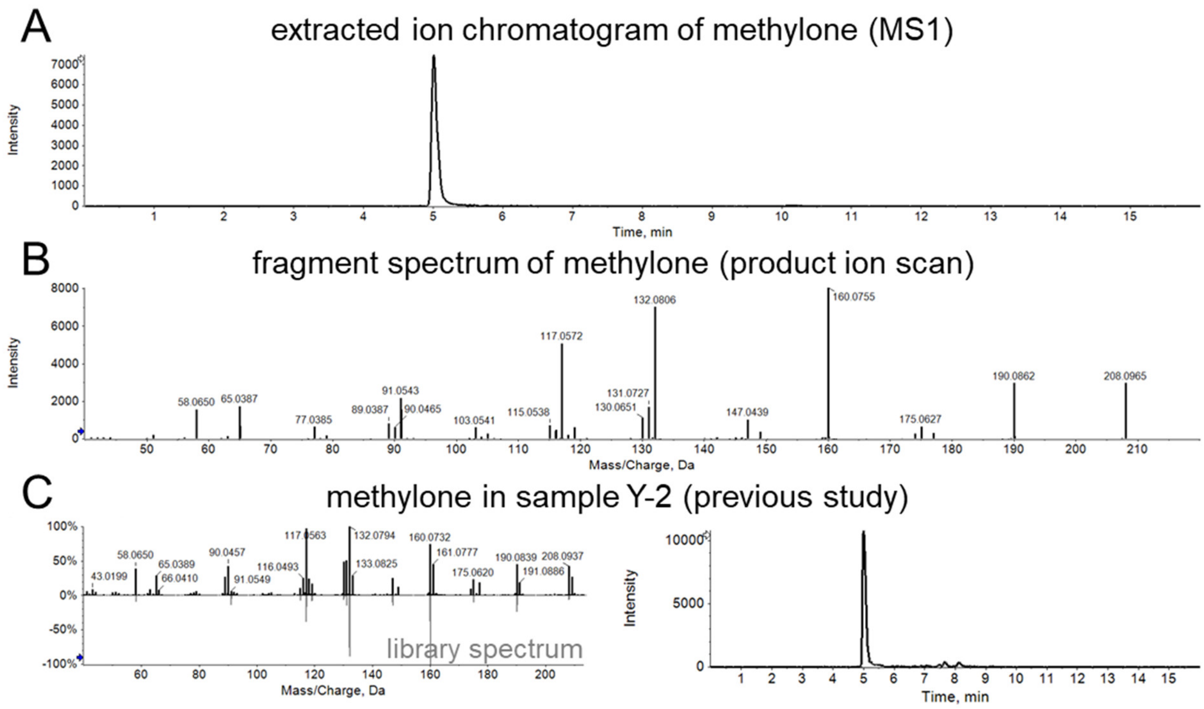


**Supplemental Figure S7.** (**A**) Extracted ion chromatogram and (**B**) fragment spectrum of methylone (Lipomed, Cat. No. MTY-1289-HC) and (**C**) spectral library matching result of the methylone-positive sample. The blue and white arrows on the y-axes indicate thresholds for presenting m/z values.

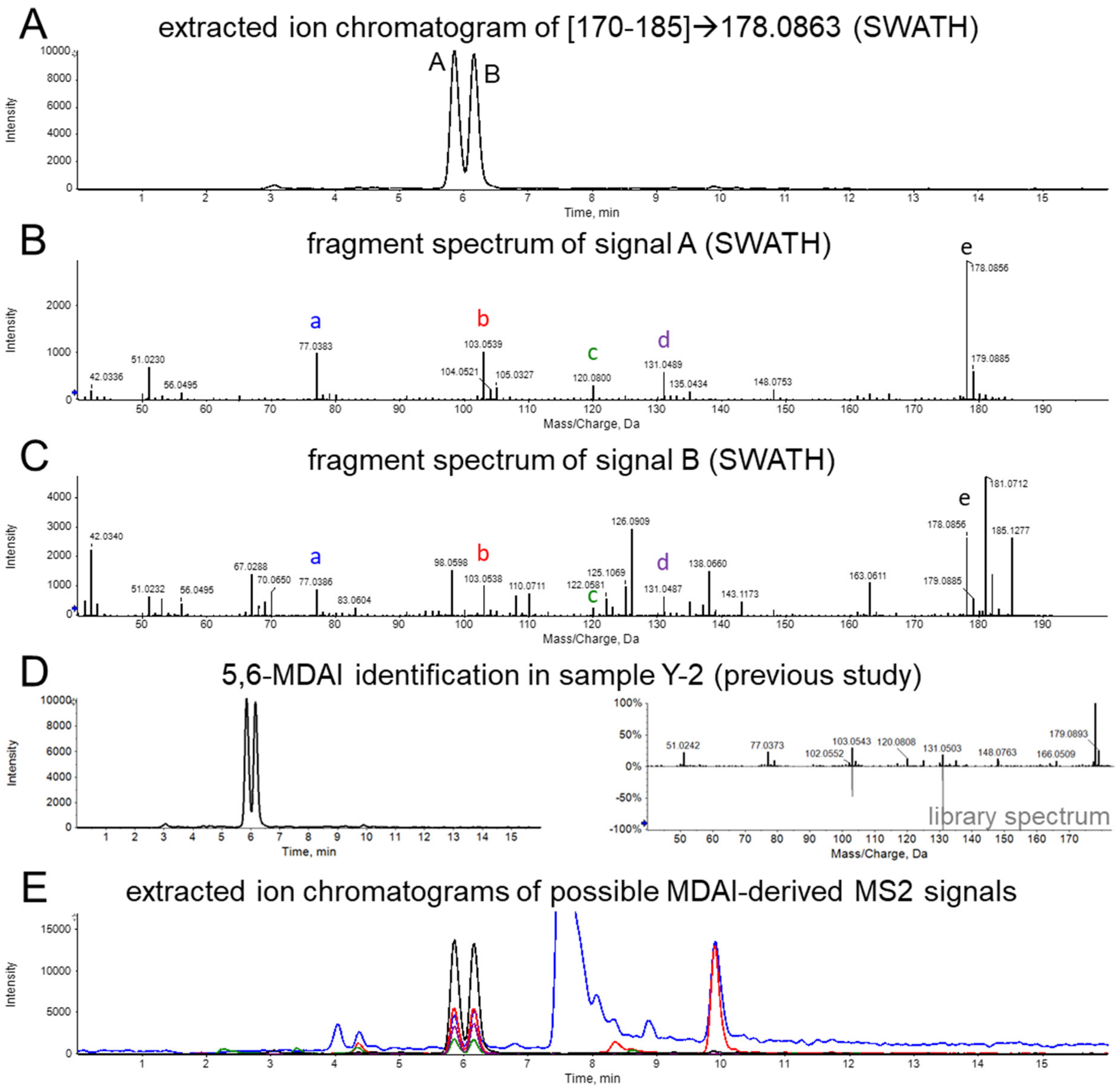


**Supplemental Figure S8.** (**A**) Extracted ion chromatogram and (**B**) fragment spectra (**C**) of two possible methylenedioxy-2-aminoindanes (MDAI) as well as (**D**) spectral library matching result for 5,6-MDAI, and (**E**) extracted ion chromatograms of presumed MDAI-derived fragments, as observed in the 5,6-MDAI-positive sample. The blue and white arrows on the y-axes indicate thresholds for presenting m/z values.

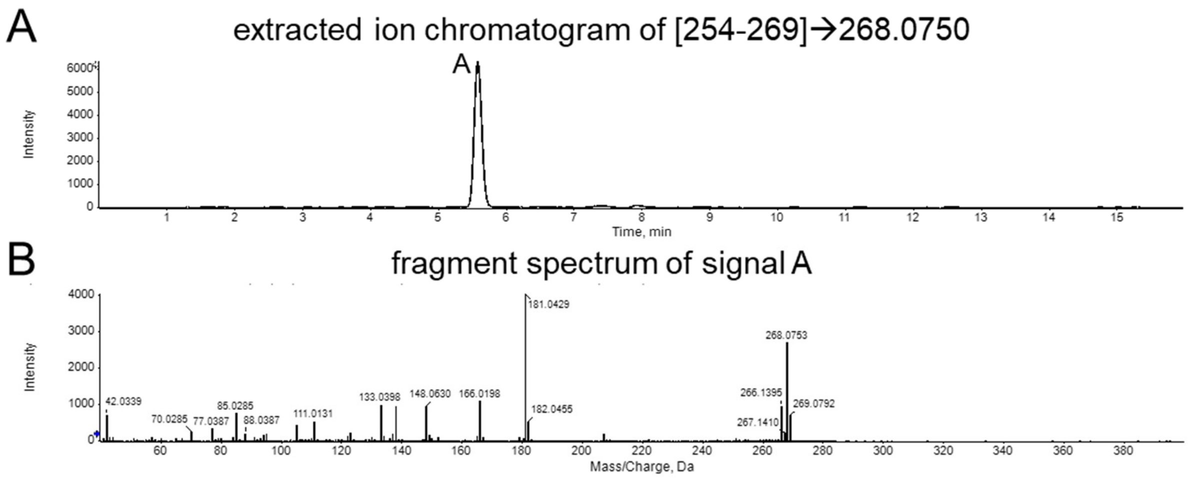


**Supplemental Figure S9**. Exemplary (**A**) extracted ion chromatogram and (**B**) fragment spectrum of a possible cysteine metabolite of omeprazole. The blue and white arrows on the y-axes indicate thresholds for presenting m/z values.

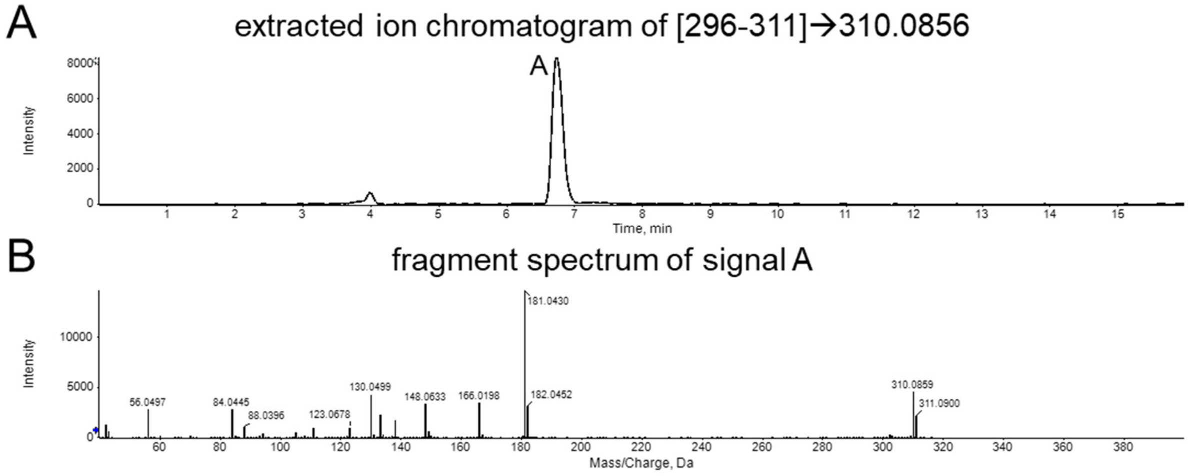


**Supplemental Figure S10**. Exemplary (**A**) extracted ion chromatogram and (**B**) fragment spectrum of a possible mercapturate metabolite of omeprazole. The blue and white arrows on the y-axes indicate thresholds for presenting m/z values.

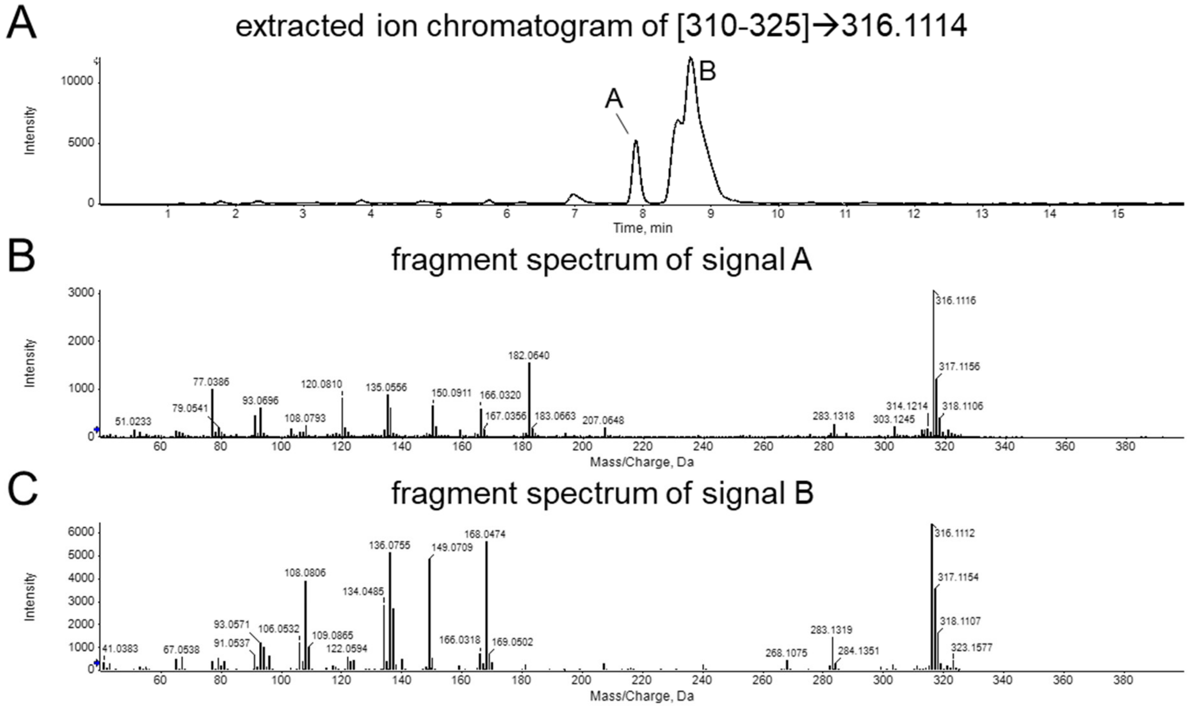


**Supplemental Figure S11**. Exemplary (**A**) extracted ion chromatogram and (**B**, **C**) fragment spectra of possible demethylated and dehydroxylated metabolites of omeprazole. The blue and white arrows on the y-axes indicate thresholds for presenting m/z values.

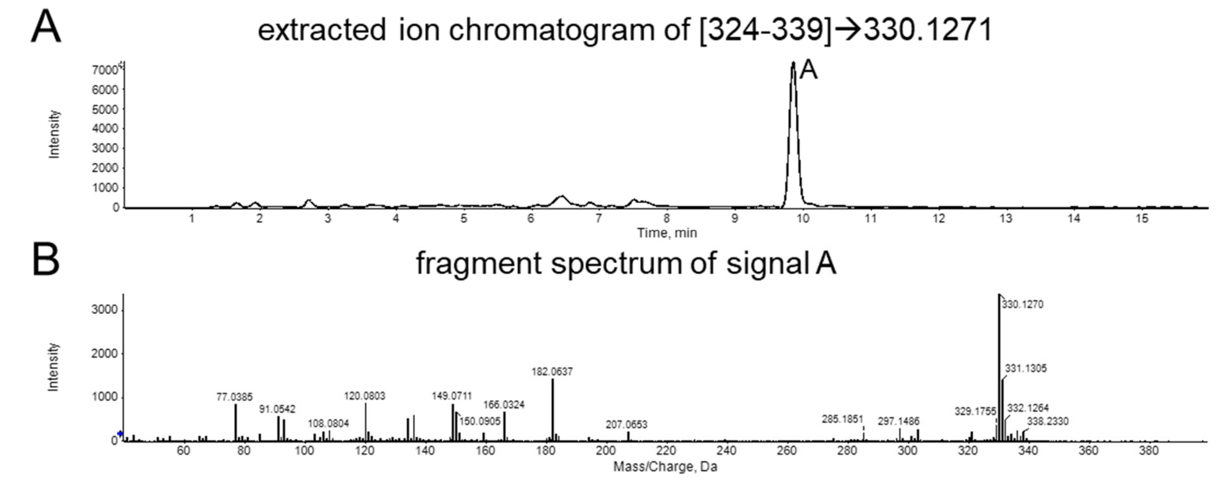


**Supplemental Figure S12**. Exemplary (**A**) extracted ion chromatogram and (**B**) fragment spectrum of a possible dehydroxylated metabolite of omeprazole. The blue and white arrows on the y-axes indicate thresholds for presenting m/z values.

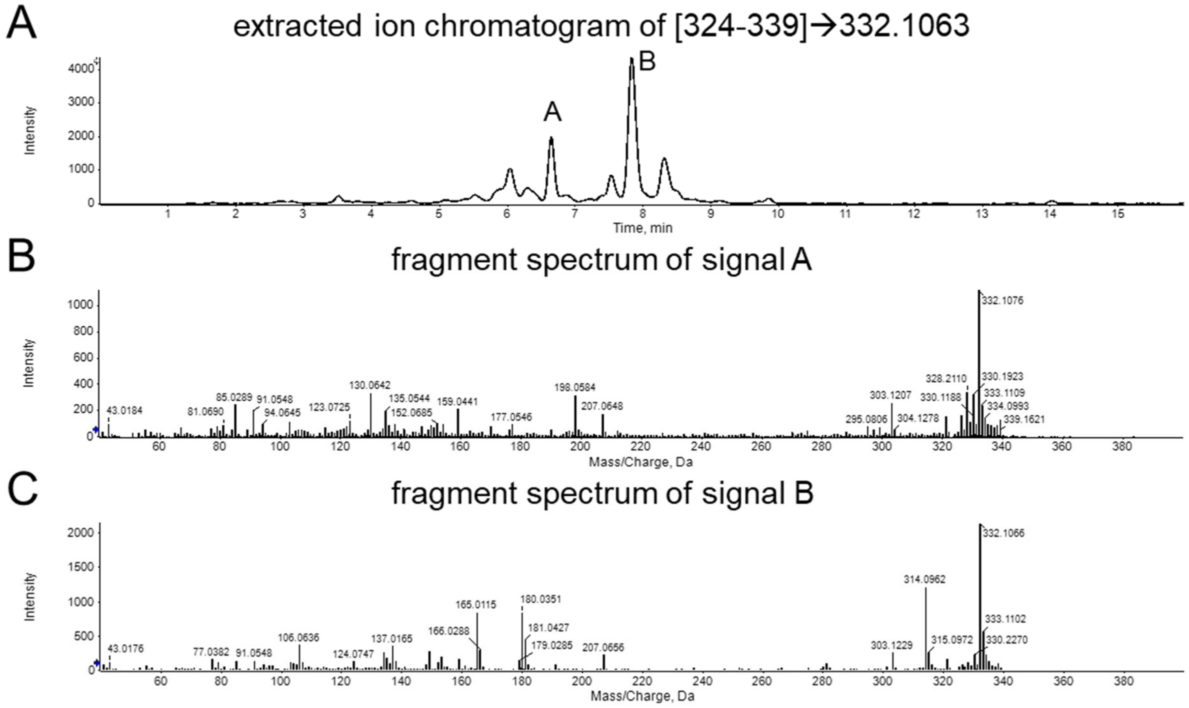


**Supplemental Figure S13**. Exemplary (**A**) extracted ion chromatogram and (**B**, **C**) fragment spectra of possible demethylated metabolites of omeprazole. The blue and white arrows on the y-axes indicate thresholds for presenting m/z values.

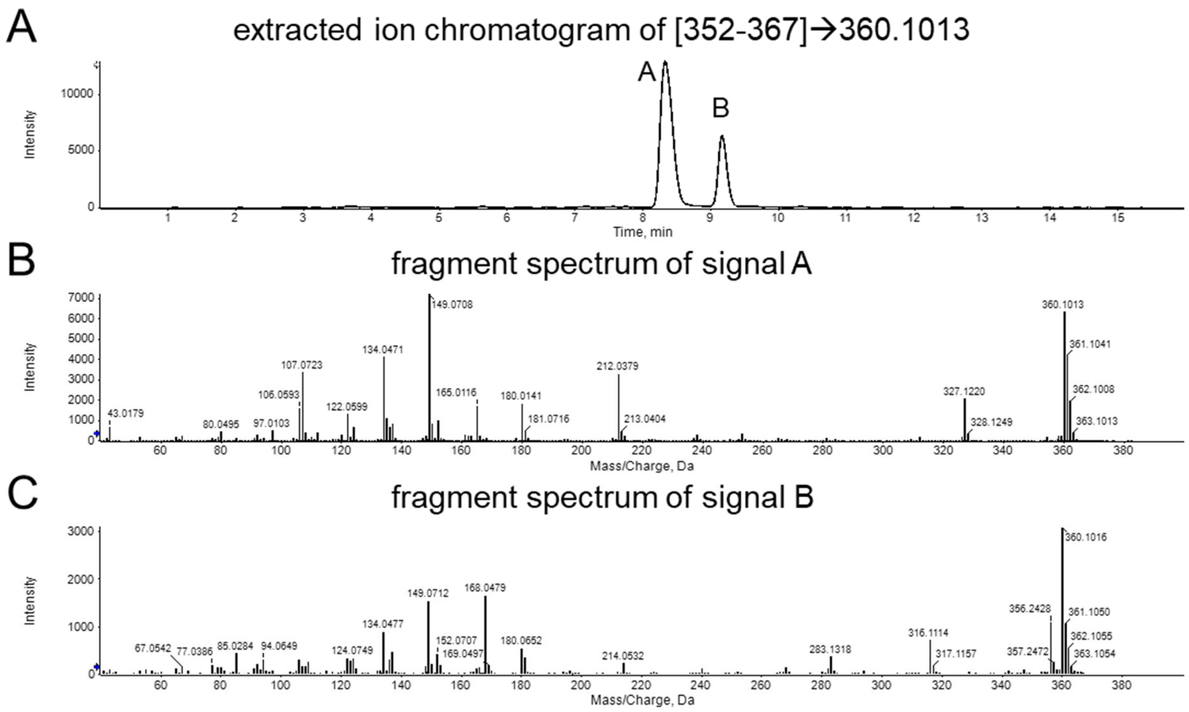


**Supplemental Figure S14**. Exemplary (**A**) extracted ion chromatogram and (**B**, **C**) fragment spectra of possible dehydroxylated and carboxylated metabolites of omeprazole. The blue and white arrows on the y-axes indicate thresholds for presenting m/z values.

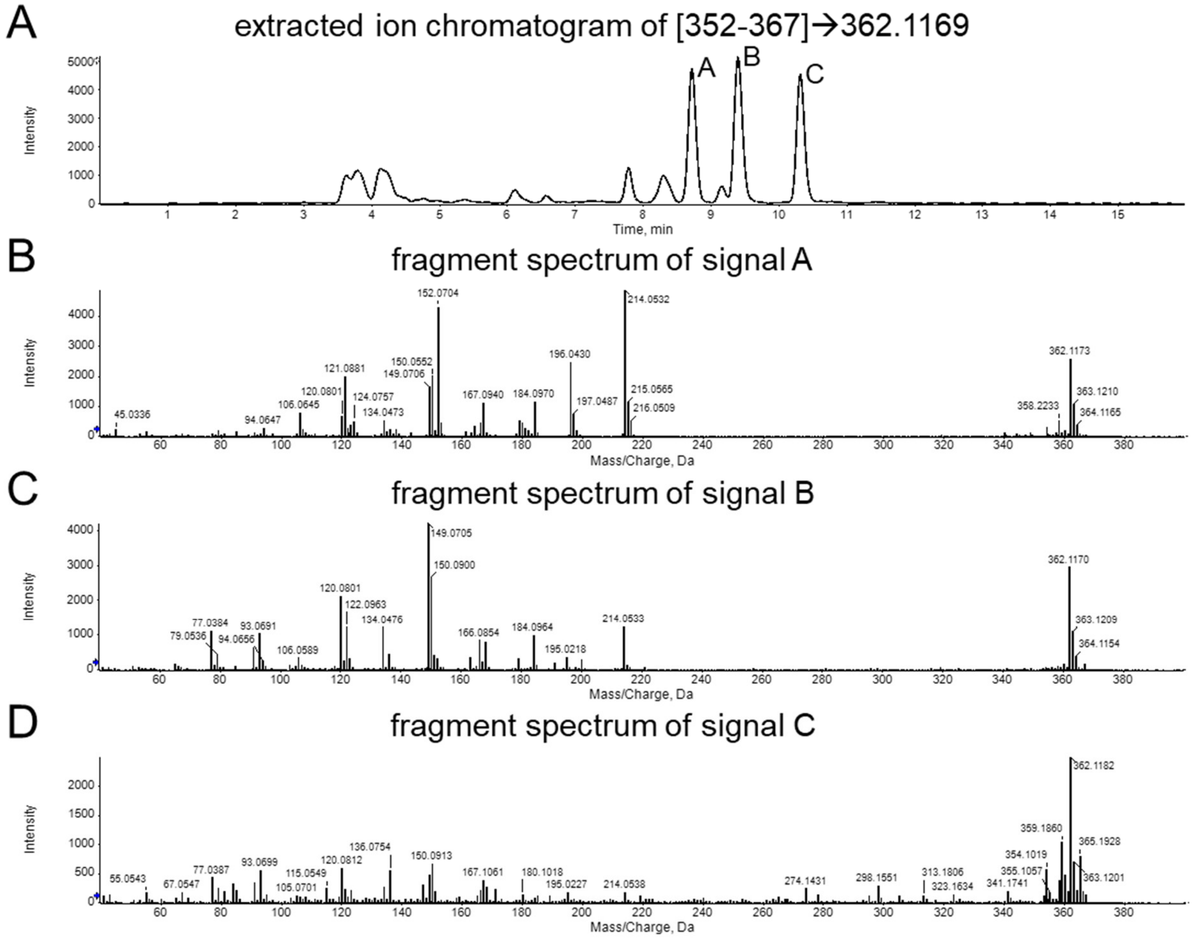


**Supplemental Figure S15**. Exemplary (**A**) extracted ion chromatogram and (**B**, **C**, **D**) fragment spectra of possible hydroxylated metabolites of omeprazole. The blue and white arrows on the y-axes indicate thresholds for presenting m/z values.

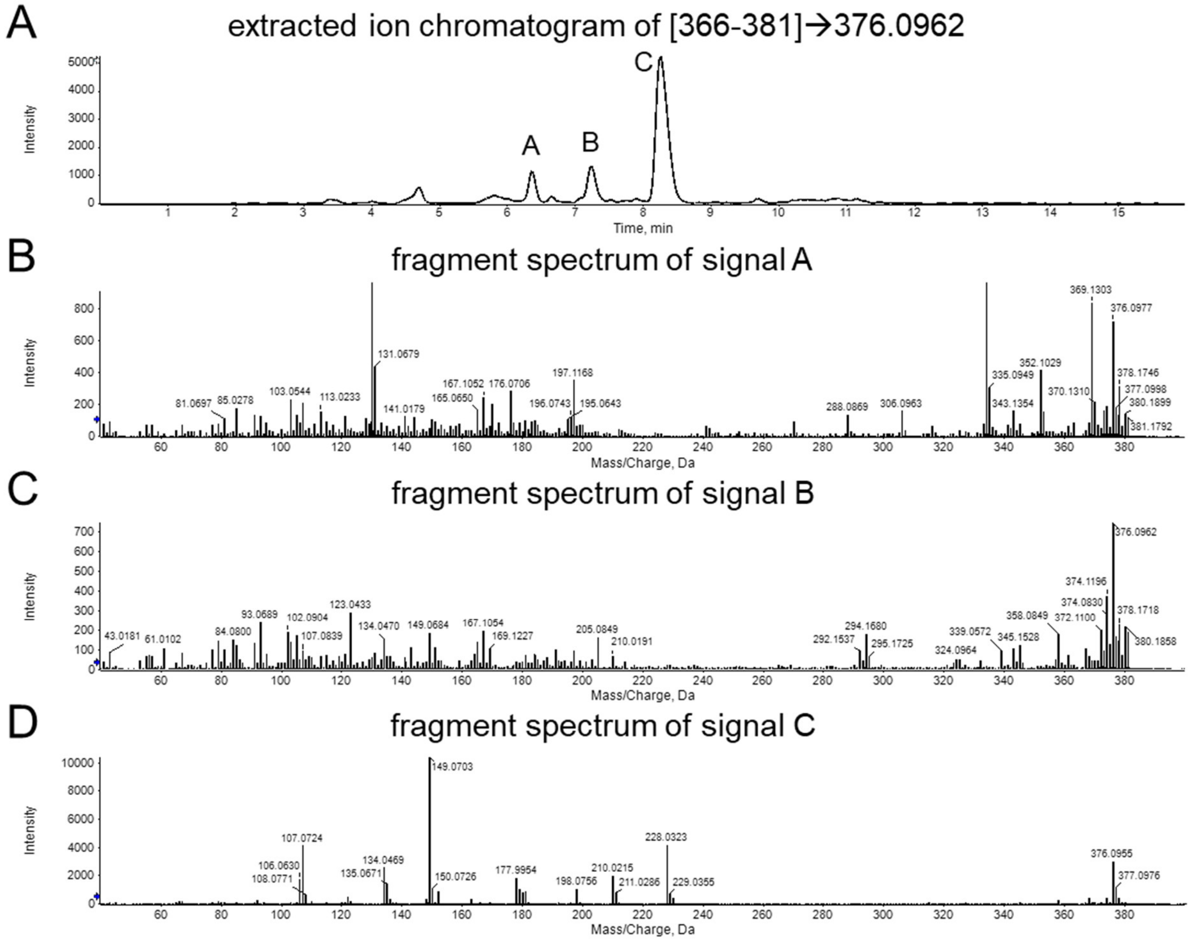


**Supplemental Figure S16**. Exemplary (**A**) extracted ion chromatogram and (**B**, **C**, **D**) fragment spectra of possible carboxylated metabolites of omeprazole. The blue and white arrows on the y-axes indicate thresholds for presenting m/z values.

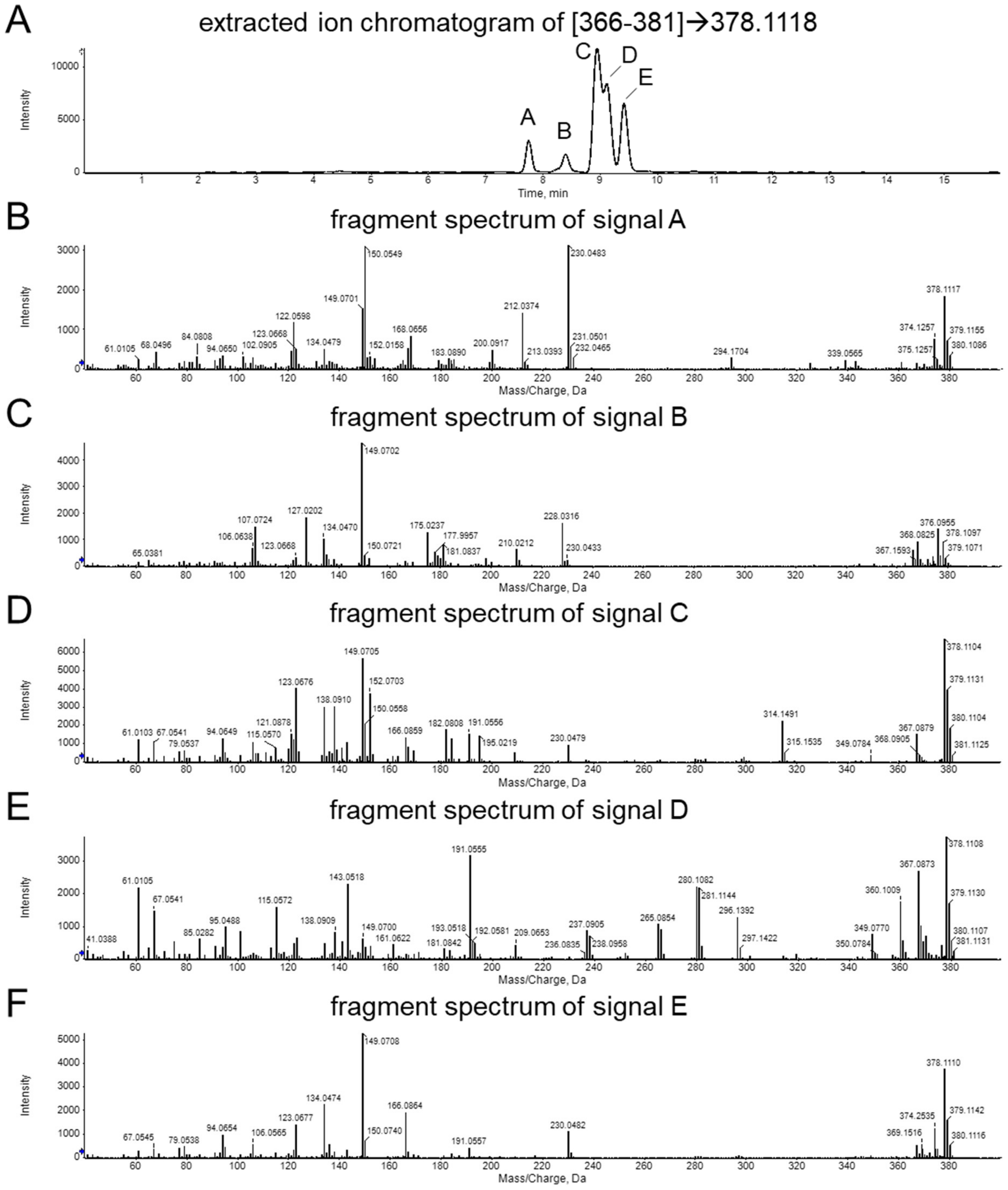


**Supplemental Figure S17**. Exemplary (**A**) extracted ion chromatogram and (**B**, **C**, **D**, **E**, **F**) fragment spectra of possible dihydroxylated metabolites of omeprazole. The blue and white arrows on the y-axes indicate thresholds for presenting m/z values.

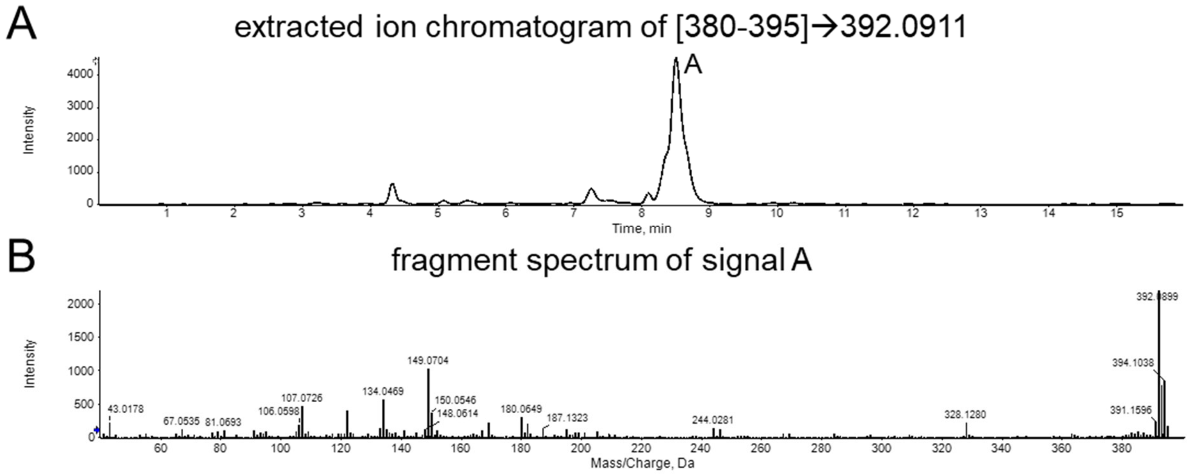


**Supplemental Figure S18**. Exemplary (**A**) extracted ion chromatogram and (**B**) fragment spectrum of a possible hydroxylated and carboxylated metabolite of omeprazole. The blue and white arrows on the y-axes indicate thresholds for presenting m/z values.

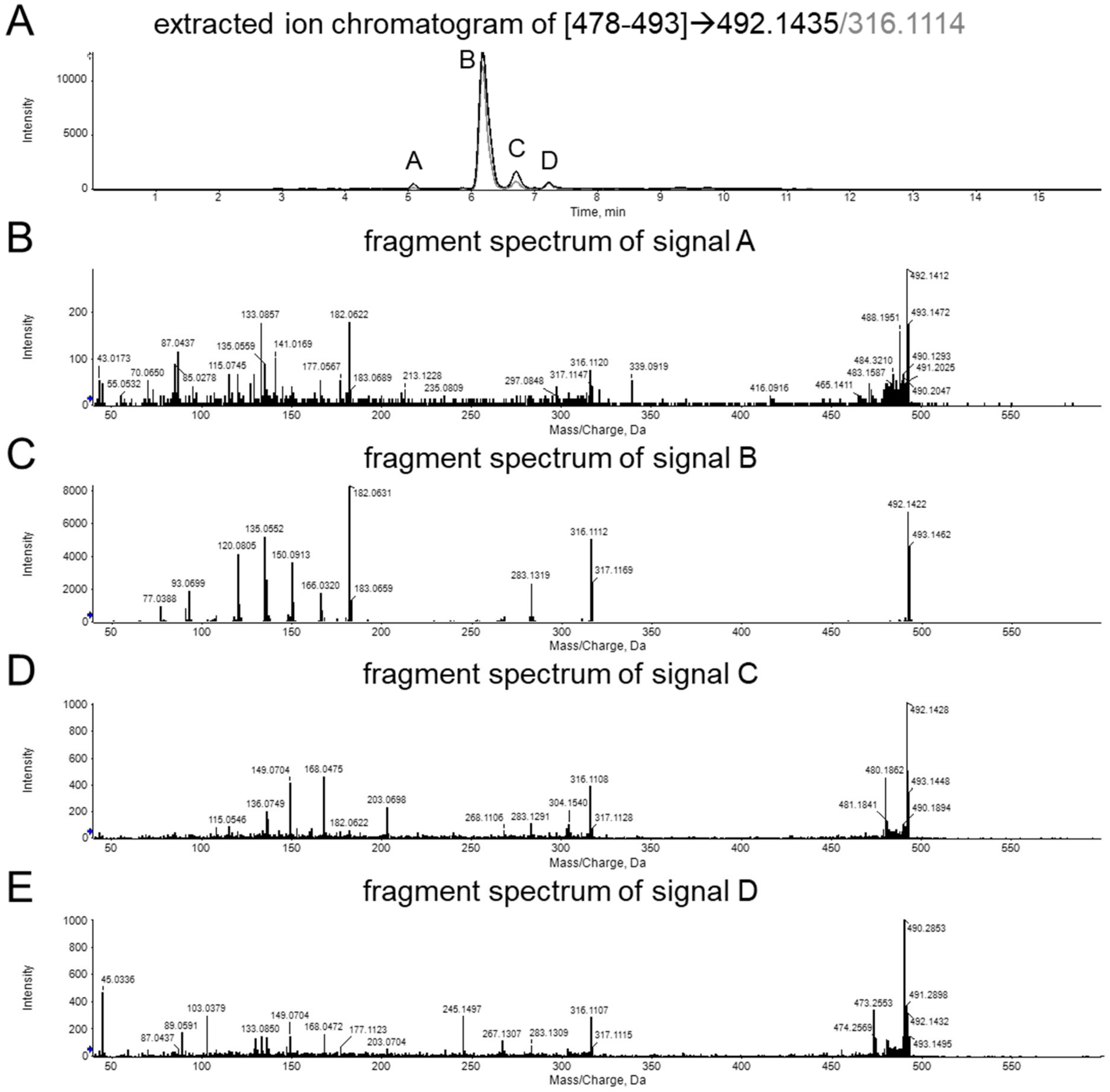


**Supplemental Figure S19**. Exemplary (**A**) extracted ion chromatogram and (**B**, **C**, **D**, **E**) fragment spectra of possible demethylated, dehydroxylated, and glucuronidated metabolites of omeprazole. The blue and white arrows on the y-axes indicate thresholds for presenting m/z values.

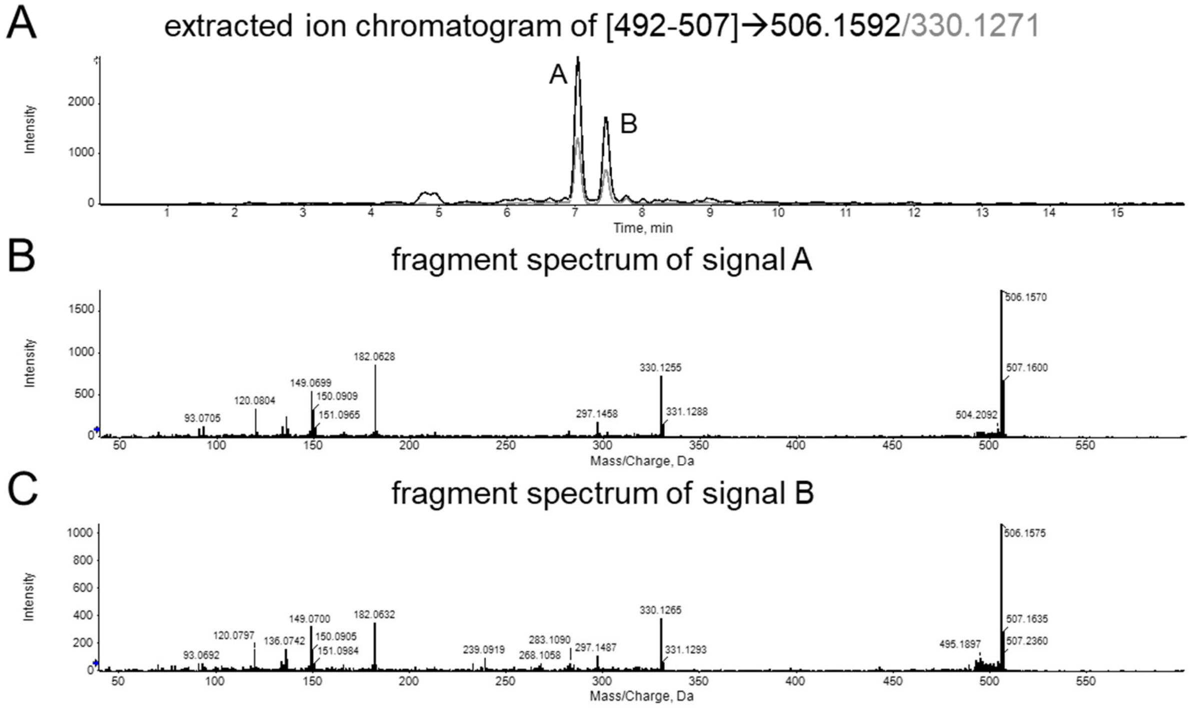


**Supplemental Figure S20**. Exemplary (**A**) extracted ion chromatogram and (**B**, **C**) fragment spectra of possible dehydroxylated and glucuronidated metabolites of omeprazole. The blue and white arrows on the y-axes indicate thresholds for presenting m/z values.

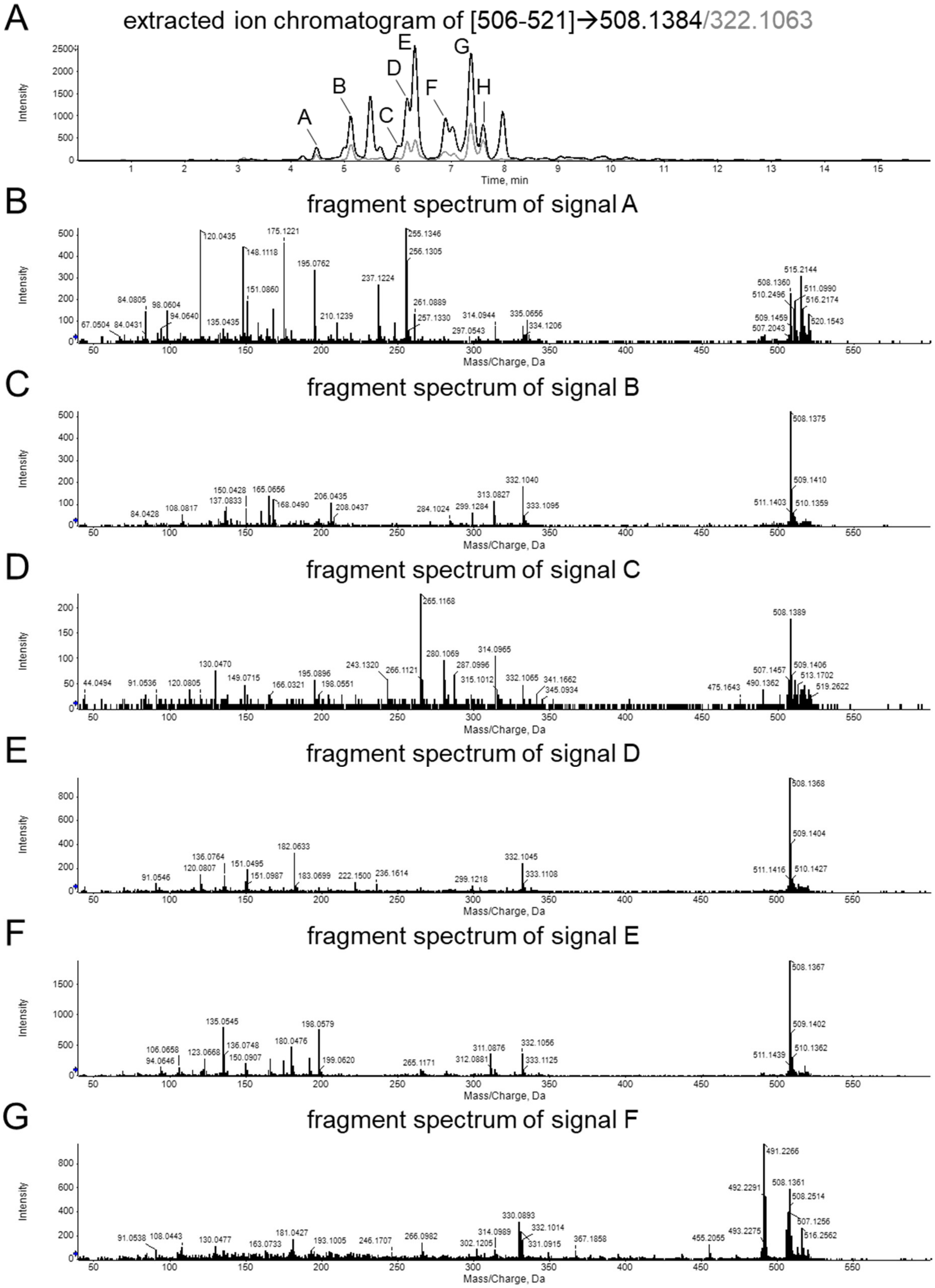


**Supplemental Figure S21**. Exemplary (**A**) extracted ion chromatogram and (**B**, **C**, **D**, **E**, **F**, **G**, **H**, **I**) fragment spectra of possible demethylated and glucuronidated metabolites of omeprazole. The blue and white arrows on the y-axes indicate thresholds for presenting m/z values.

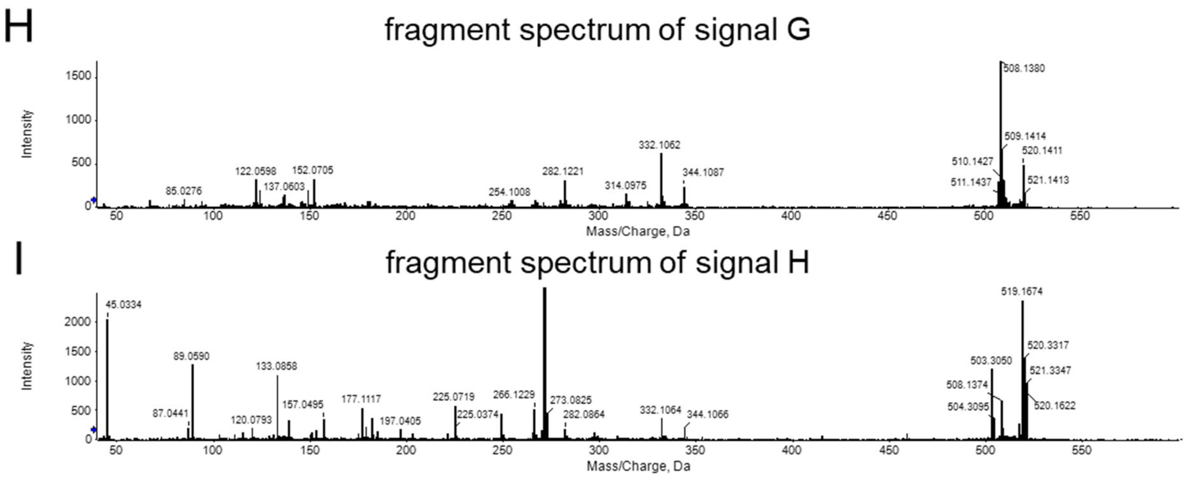


**Supplemental Figure S21**. *Cont*.

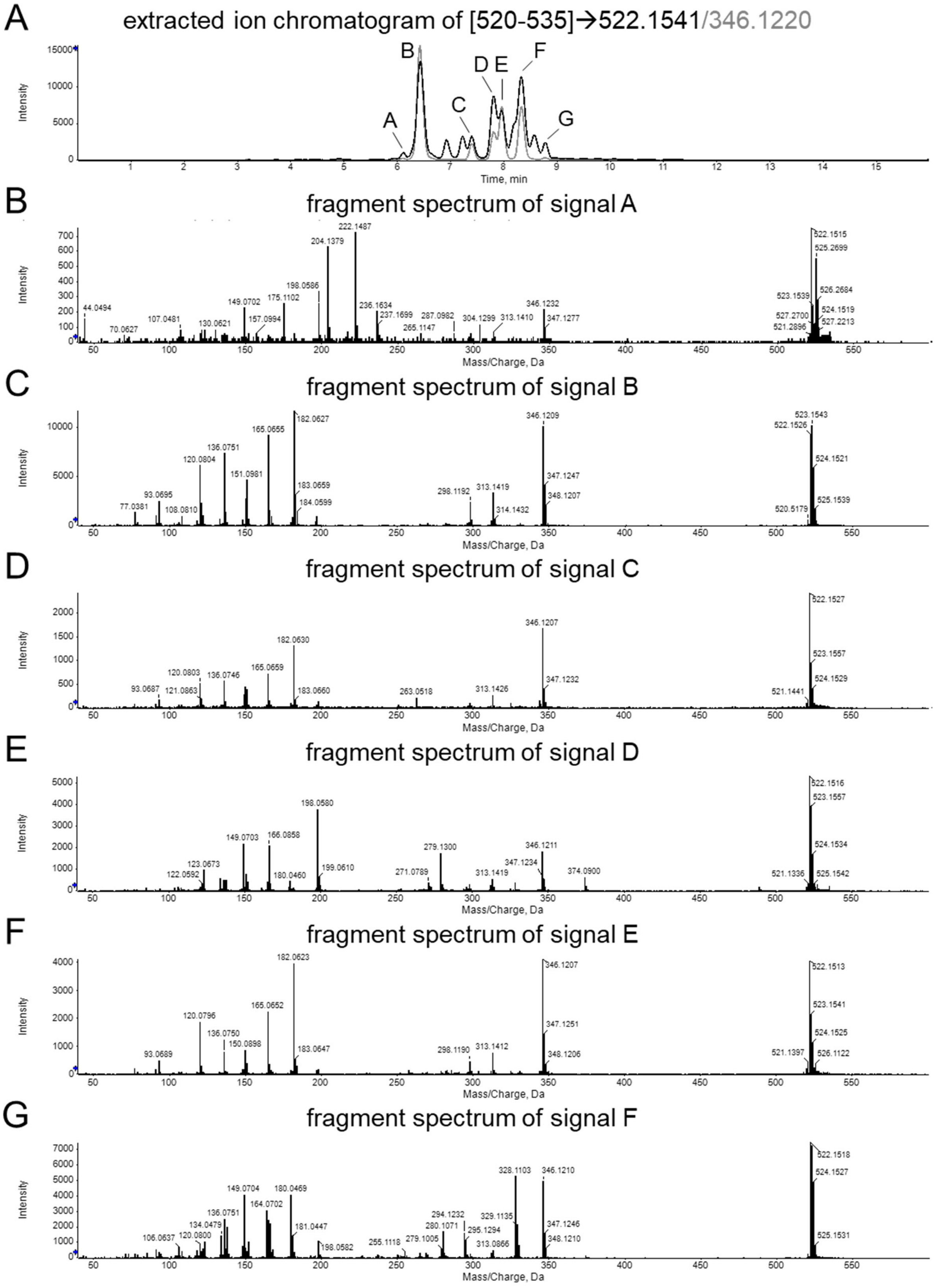


**Supplemental Figure S22**. Exemplary (**A**) extracted ion chromatogram and (**B, C, D, E, F, G, H**) fragment spectra of possible glucuronidated metabolites of omeprazole. The blue arrows on the y-axes indicate thresholds for presenting m/z values.

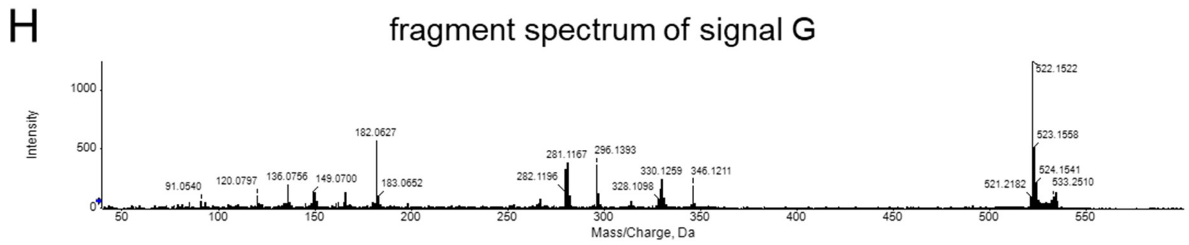


**Supplemental Figure S22**. *Cont*.

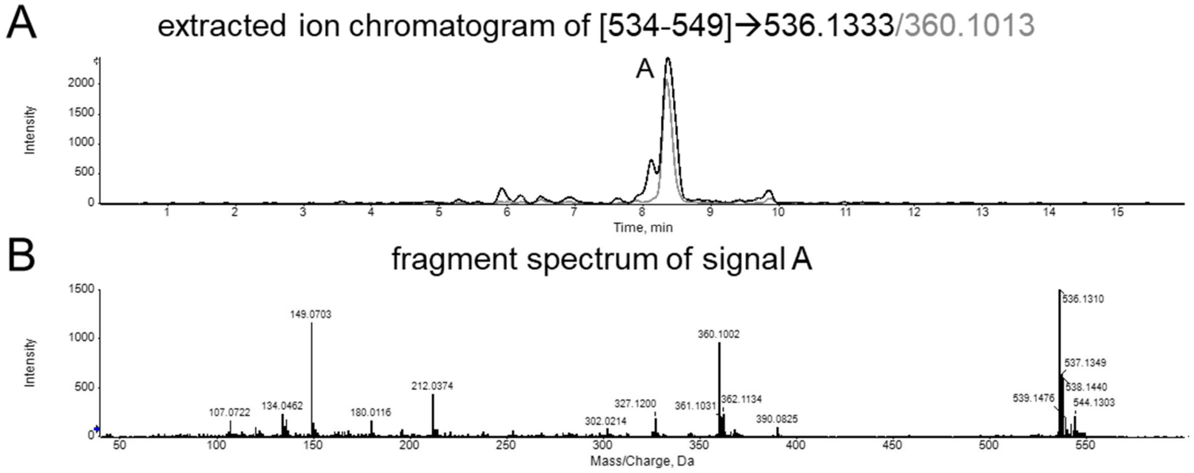


**Supplemental Figure S23**. Exemplary (**A**) extracted ion chromatogram and (**B**) fragment spectrum of a possible dehydroxylated, carboxylated, and glucuronidated metabolite of omeprazole. The blue and white arrows on the y-axes indicate thresholds for presenting m/z values.

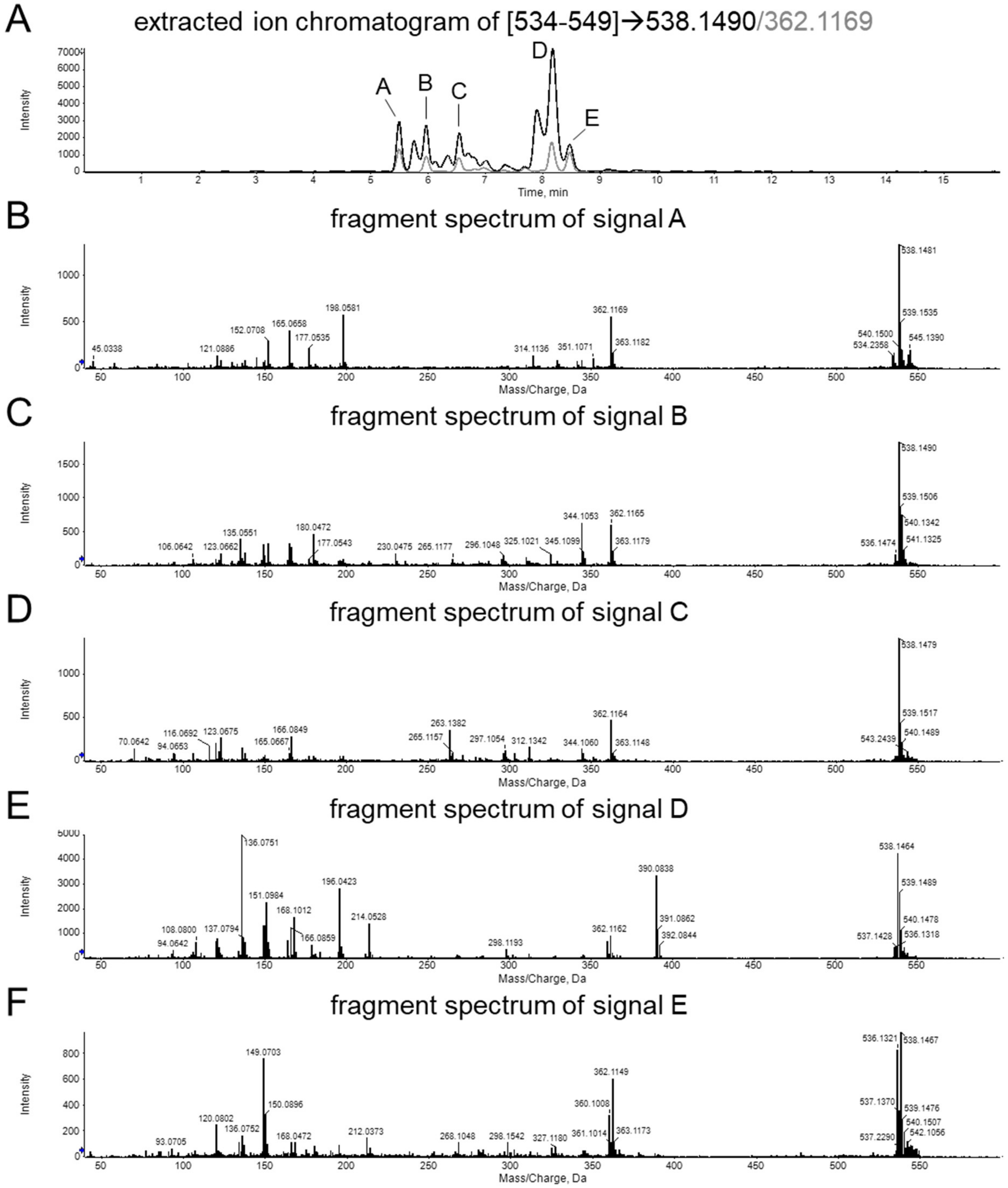


**Supplemental Figure S24**. Exemplary (**A**) extracted ion chromatogram and (**B**, **C**, **D**, **E**, **F**) fragment spectra of possible hydroxylated and glucuronidated metabolites of omeprazole. The blue and white arrows on the y-axes indicate thresholds for presenting m/z values.

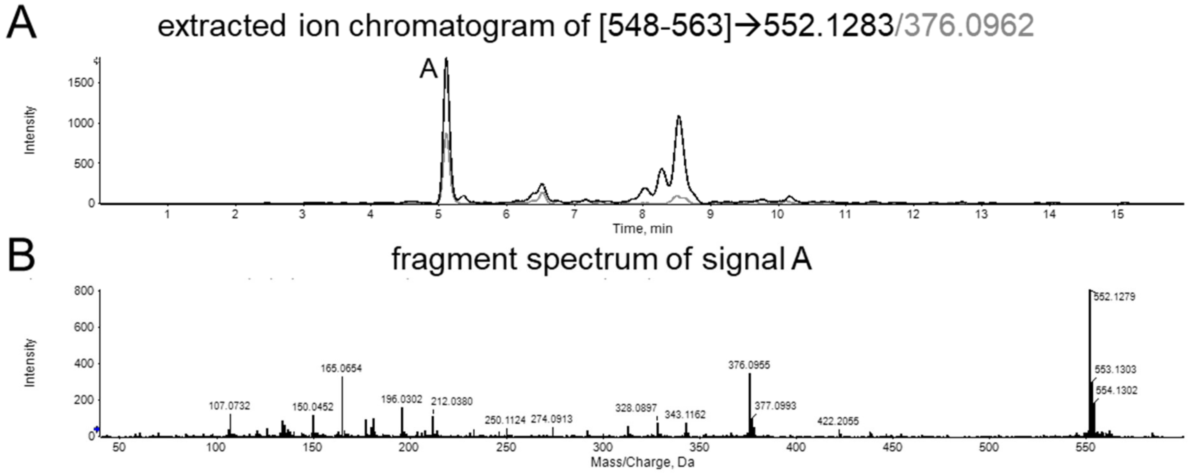


**Supplemental Figure S25**. Exemplary (**A**) extracted ion chromatogram and (**B**) fragment spectrum of a possible carboxylated and glucuronidated metabolite of omeprazole. The blue and white arrows on the y-axes indicate thresholds for presenting m/z values.

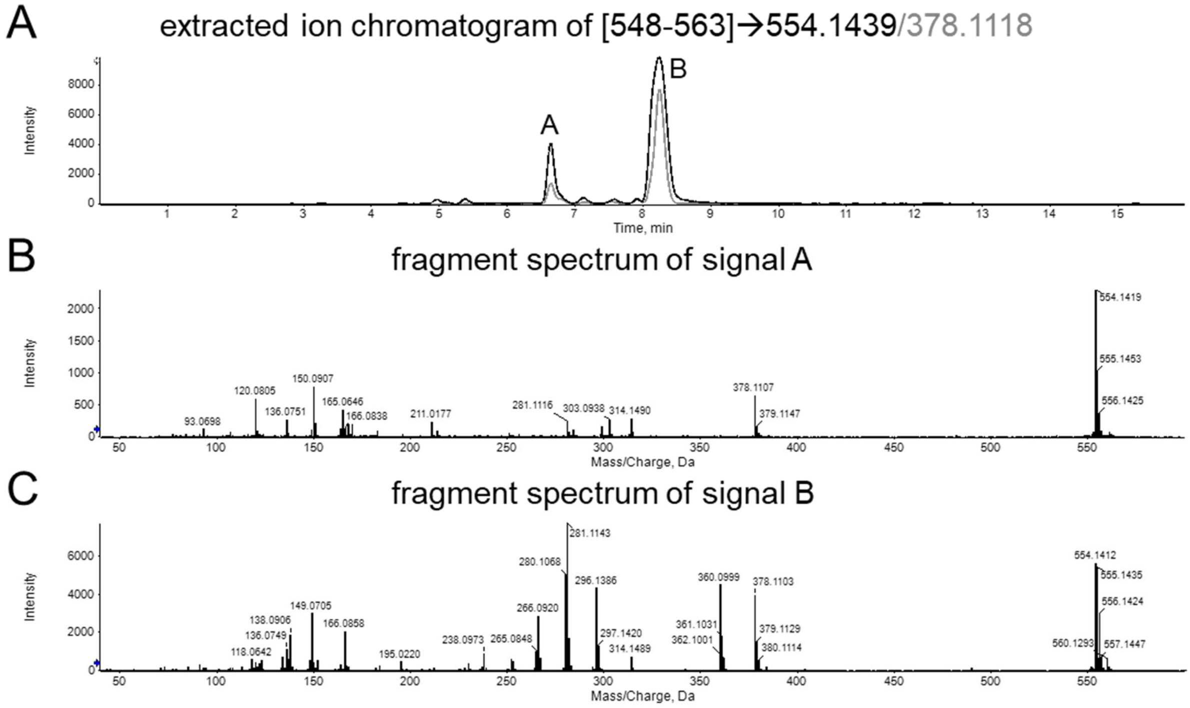


**Supplemental Figure S26**. Exemplary (**A**) extracted ion chromatogram and (**B**, **C**) fragment spectra of possible dihydroxylated and glucuronidated metabolites of omeprazole. The blue and white arrows on the y-axes indicate thresholds for presenting m/z values.
